# Generation and characterization of a bivalent protein boost for future clinical trials: HIV-1 subtypes CR01_AE and B gp120 antigens with a potent adjuvant

**DOI:** 10.1371/journal.pone.0194266

**Published:** 2018-04-26

**Authors:** Yingxia Wen, Hung V. Trinh, Christine E. Linton, Chiara Tani, Nathalie Norais, DeeAnn Martinez-Guzman, Priyanka Ramesh, Yide Sun, Frank Situ, Selen Karaca-Griffin, Christopher Hamlin, Sayali Onkar, Sai Tian, Susan Hilt, Padma Malyala, Rushit Lodaya, Ning Li, Gillis Otten, Giuseppe Palladino, Kristian Friedrich, Yukti Aggarwal, Celia LaBranche, Ryan Duffy, Xiaoying Shen, Georgia D. Tomaras, David C. Montefiori, William Fulp, Raphael Gottardo, Brian Burke, Jeffrey B. Ulmer, Susan Zolla-Pazner, Hua-Xin Liao, Barton F. Haynes, Nelson L. Michael, Jerome H. Kim, Mangala Rao, Robert J. O’Connell, Andrea Carfi, Susan W. Barnett

**Affiliations:** 1 Novartis Vaccines and Diagnostics, Cambridge, MA, United States of America; 2 US Military HIV Research Program, Walter Reed Army Institute of Research, Silver Spring, MD, United States of America; 3 Henry Jackson Foundation for the Advancement of Military Medicine, Silver Spring, MD, United States of America; 4 GSK, Rockville, MD, United States of America; 5 GSK, Siena, Italy; 6 Department of Surgery, Duke University Medical Center, Durham, NC, United States of America; 7 Duke Human Vaccine Institute, Duke University, Durham, NC, United States of America; 8 Vaccine and Infectious Disease Division, Fred Hutchinson Cancer Research Center, Seattle, WA, United States of America; 9 Icahn School of Medicine at Mount Sinai, New York, NY, United States of America; 10 Biomedine Institute, College of Life Science, Jinan University, Guangzhou, China; 11 Armed Forces Research Institute of Medical Sciences, Bangkok, Thailand; Emory University School of Medicine, UNITED STATES

## Abstract

The RV144 Phase III clinical trial with ALVAC-HIV prime and AIDSVAX B/E subtypes CRF01_AE (A244) and B (MN) gp120 boost vaccine regime in Thailand provided a foundation for the future development of improved vaccine strategies that may afford protection against the human immunodeficiency virus type 1 (HIV-1). Results from this trial showed that immune responses directed against specific regions V1V2 of the viral envelope (Env) glycoprotein gp120 of HIV-1, were inversely correlated to the risk of HIV-1 infection. Due to the low production of gp120 proteins in CHO cells (2–20 mg/L), cleavage sites in V1V2 loops (A244) and V3 loop (MN) causing heterogeneous antigen products, it was an urgent need to generate CHO cells harboring A244 gp120 with high production yields and an additional, homogenous and uncleaved subtype B gp120 protein to replace MN used in RV144 for the future clinical trials. Here we describe the generation of Chinese Hamster Ovary (CHO) cell lines stably expressing vaccine HIV-1 Env antigens for these purposes: one expressing an HIV-1 subtype CRF01_AE A244 Env gp120 protein (A244.AE) and one expressing an HIV-1 subtype B 6240 Env gp120 protein (6240.B) suitable for possible future manufacturing of Phase I clinical trial materials with cell culture expression levels of over 100 mg/L. The antigenic profiles of the molecules were elucidated by comprehensive approaches including analysis with a panel of well-characterized monoclonal antibodies recognizing critical epitopes using Biacore and ELISA, and glycosylation analysis by mass spectrometry, which confirmed previously identified glycosylation sites and revealed unknown sites of O-linked and N-linked glycosylations at non-consensus motifs. Overall, the vaccines given with MF59 adjuvant induced higher and more rapid antibody (Ab) responses as well as higher Ab avidity than groups given with aluminum hydroxide. Also, bivalent proteins (A244.AE and 6240.B) formulated with MF59 elicited distinct V2-specific Abs to the epitope previously shown to correlate with decreased risk of HIV-1 infection in the RV144 trial. All together, these results provide critical information allowing the consideration of these candidate gp120 proteins for future clinical evaluations in combination with a potent adjuvant.

## Introduction

In Thailand, HIV-1/AIDS was first reported in the 1980s, and since then, over one million Thai adults have become infected, with nearly 600,000 having died from AIDS-related diseases, with an estimated 7,816 new HIV-1 infections and 20,492 AIDS related deaths in 2014 alone [1]. With HIV-1 prevalence in adults at 1.1%, Thailand continues to have one of the highest prevalence rates of reported HIV-1 in Asia [2]. Due to the enormous global economic, social, and health burdens of HIV-1/AIDS and despite the expanded availability of highly effective anti-retroviral drugs, the development of an efficacious prophylactic vaccine against HIV-1 remains a high priority, particularly among those populations hardest hit by the disease and with limited access to anti-viral drugs [3]. While subtype B is the major HIV-1 subtype in North America and Europe [4], it is also prevalent in other regions including Asia [5, 6].

The HIV-1 envelope glycoprotein (Env) is a critical target for the host immune system and is currently the major focus of the rational immunogen design for the next generation of HIV-1 vaccines. The viral Env is composed of the gp120 surface and gp41 transmembrane glycoproteins that together form trimeric structures to decorate the surface of native virions and mediate interaction with its host cell surface receptor (CD4), which occurs via the CD4 binding site (CD4bs) domain of the gp120 protein. The CD4bs and other regions of gp120 represent critical epitopes recognized by the host’s immune system and are targets for broadly reactive neutralizing antibodies (nAbs) as well as other functional antibody (Ab) responses [7–11].

RV144 was a double-blind and randomized Phase III clinical trial conducted in Thailand evaluating a prime-boost vaccine strategy for the prevention of infection and amelioration of disease course in HIV-1-negative volunteers who were at community-risk of HIV-1 infection. This trial showed 31.2% efficacy 42 months after the first immunization and 60% efficacy at 12 months [12, 13]. The vaccine regimen consisted of the Sanofi-Pasteur’s recombinant ALVAC-HIV (vCP1521) as a prime and the VaxGen (now the non-profit entity, Global Solutions for Infectious Disease or GSID) bivalent gp120 B/E (AIDSVAX B/E) as a protein boost. Although the observed efficacy from the vaccine was modest, the study showed for the first time that an HIV-1 vaccine candidate was capable of reducing HIV-1 acquisition risk, especially at early time points after vaccination, warranting further testing of similar and potentially improved vaccine regimens [13, 14].

A comprehensive immune correlates of risk analysis in RV144 revealed that Ab responses to variable loops 1 and 2 (V1/V2) on gp120 correlated inversely with infection risk [15–17], and a molecular sieve analysis showed that specific epitopes in the V2 region were subjected to immune pressure by the vaccine [18]. Moreover, plasma IgG responses to linear epitopes in V2 and V3 were found to correlate with reduced HIV-1 infection risk [19]. Further analyses of the quality and functionality of Abs demonstrated that anti-V1V2 Abs of the IgG3 subclass were associated with decreased HIV-1 risk [20] and increased polyfunctionality [21, 22] with newer evidence that IgG1 subclass Ab associated with Ab-dependent cellular phagocytosis might also play a role [23]. Propelled by these new findings, clinical vaccine development activities were mobilized through the formation of the Pox-Protein Public Private Partnership or “P5” in 2010 to follow-up on RV144 [24]. The near-term plan would be to evaluate a vaccine regime similar to the one used in RV144, but modified to target the most common HIV-1 subtype in the southern African region (subtype C). Hence, product development activities ensued to provide subtype C-specific vaccine candidates, a recombinant pox vector prime encoding an Env antigen from HIV-1_ZM96_ (subtype C) and a new bivalent protein boost composed of gp120s from subtype C, HIV-1_TV1_ and HIV-1_1086_ [25, 26]. These candidate vaccines recently showed to elicit significant anti-gp120 and V2-directed Ab responses as well as polyfunctional CD4 T cell responses as seen in the RV144 trial [27, 28], in the HVTN100 Phase I clinical trial, thus meeting the criteria set by the P5 to advance these candidates to proof of concept efficacy testing in the HVTN702 Phase IIb/III clinical trial [29].

In addition to the P5 efforts in the southern Africa region, a Product Development Advisory Group (PDAG) recommended the need to optimize protein (antigen) and adjuvant selections for the next vaccine candidates in Thailand. Because two gp120 proteins, one from subtype B and one from subtype CRF01_AE were used in the RV144 trial, the group decided to proceed with a similar bivalent composition of monomeric gp120. Importantly, the group also considered if the gp120 proteins used in RV144 (derived from subtype B, HIV-1_MN_, and subtype CRF01_AE, HIV-1_A244_) should be substituted with gp120s from other strains. An evaluation of the antigenicity of these proteins demonstrated that the V2 loop was better exposed on the A244 gp120 Env compared to MN gp120 when assessed with 697-D mAb that recognizes a conformational V2 epitope [30, 31]. Moreover, the immune correlates of risk analysis demonstrated that IgG responses to V2 MN did not correlate with HIV infection risk [15] and that the linear V2 IgG responses that inversely correlated with HIV infection risk were to a linear subtype AE sequence and not to subtype B [19]. These data suggested that the MN immunogen did not significantly contribute to induction of the V2 IgG immune correlate of risk in RV144. However, the V3 Ab responses in RV144 elicited by the subtype B gp120 were later shown to correlate with reduced risk of the HIV-1 infection [19, 32]. Given the initial intent (in the 1990s) to cover both HIV-1 subtypes CRF01_AE in Thailand and B in South East Asia, China, North America and Europe, the CRF01_AE (A244) was chosen as a predominantly circulating strain in Thai heterosexual and MSM populations, and the selection of a new subtype B gp120 candidate was proposed. This decision was also informed by the intention of the U.S. Army to address the most commonly observed HIV-1 subtype in U.S. service members, which has been subtype B, for possible use of such a vaccine to protect this population. In addition, the PDAG also recommended the evaluation of an alternative adjuvant system, such as the Novartis proprietary oil-in water emulsion MF59 [33–36] or others, to potentially provide more potent and durable protective immunity than that which had been seen with the aluminum hydroxide (AH) formulation used in the RV144 trial. The MF59 adjuvant is now being evaluated in the HVTN100/702 clinical trials [37].

To aid selection of the gp120 antigens, the Envs from various strains were evaluated for antigenicity and immunogenicity, ease of expression, and ability to bind the unmutated ancestors or intermediate ancestor Abs of clonal lineages that would ideally be induced by the candidate vaccine [38–40] resulting in the selection of HIV-1 subtype B 6240 gp120 and the RV144 AE immunogen A244 gp120 as potential clinical candidates pending results of the work described here. Among the transmitted founder envelope proteins evaluated, HIV-1 subtype B 6240 gp120 was among one of the best candidates for exposure of the CD4 binding site, CCR5 binding site, glycan, and V1V2 conformational regions [40]. Another factor considered was that VaxGen-produced gp120 antigens in RV144 had a herpes simplex virus (HSV) gD peptide tag and an 11 amino acid deletion at the gp120 N-terminus [41], which was initially thought to contribute to enhanced exposure of critical epitopes and induction of protective Ab responses in RV144-vaccinated subjects. It was later shown that the gD tag itself did not contribute, but that the deletion of hydrophobic N-terminal residues of the Env protein was sufficient to achieve the desired antigenicity and immunogenicity of the A244 gp120 [31]. Due to the low cell culture expression levels (2–20 mg/L with stable CHO cell lines harboring A244 gp120 protein used in RV144), there was a clear need to improve the production levels and optimize purification methods to prevent host endo-proteases cleavage observed within the critical antigenic V1V2 loop region. In this study, produciton levels of A244 are dramatically increased and protease cleavage of A244 in the V1V2 region is prevented. Moreover, to avoid the degradation of the Env gp120 protein [42], a proteolytic cleavage site in the V3 region of B.6240 was mutated and show to successfully prevent cleavage.

Here we described the generation of stable CHO cell lines, one expressing the HIV-1 subtype B 6240 gp120 (6240.B) protein and one expressing the subtype CRF01_AE A244 gp120 protein (A244.AE) at sufficient levels for future development and manufacturing. We reported results of a comprehensive evaluation of antigenicity of the purified proteins using a panel of well-characterized HIV-1-specific mAbs, glycosylation mapping and profile analyses by mass spectrometry, and an evaluation of immunogenicity using monovalent and bivalent combinations of the gp120s in guinea pigs with either AH, the adjuvant used in RV144, or MF59, the adjuvant being evaluated in the HVTN100/702 clinical trials, the latter with the goal of potentially improving the titer and the kinetics of the immune responses. Since RV144 provided a baseline for potentially protective efficacy based on the Ab responses, we focused on evaluating Ab responses for the HIV-1 vaccine candidates in this study.

## Materials & methods

### Plasmids, sCD4, and monoclonal antibodies

Codon-optimized A244.AE and 6240.B gp120 coding sequences with 11 amino acids deleted from N-terminus (Δ11 gp120) were generated synthetically using a heterologous signal leader sequence (Life Technologies). The expression cassettes were cloned into a plasmid vector containing two selectable markers, a Neomycin resistance marker and mutated dihydrofolate reductase gene (DHFR), for Chinese Hamster Ovary (CHO-K1) stable cell line development. The CHO-K1 was obtained from American Type Culture Collection (ATCC).

CD4-IgG2 (sCD4) was purchased from Progenics Pharmaceuticals. The monoclonal antibodies (mAbs) CH01 [38], CH58 and CH59 [43] were generously provided by Drs. Larry Liao and Barton Haynes (Duke University, Durham, NC). The mAbs 2219, 2557, 697, 830A, 1393 and 2158 were generously provided by Dr. Susan Zolla-Pazner (Icahn School of Medicine at Mount Sinai, NY, NY). Other mAbs were generated by cloning the published sequences of VL and VH chains (obtained from PDB database) into an Ab-expression vector backbone containing the human CL and CH chains. The plasmids were transfected into HEK 293T cells and the expressed mAb-containing culture supernatants were purified using Protein A affinity chromatography (GE Healthcare Bio-Sciences). The HEK 293T cells were purchased from Life Technologies.

### Generation of stable CHO cell lines containing A244.AE or 6240.B gp120 proteins

Expression plasmids were transfected into the Novartis proprietary CHO K1PD parental cell line, which was derived originally from CHO-K1 (ATCC), by electroporation. Transfected CHO cell pools were subjected to two sequential selections, 0.8 mg/ml geneticin (G418) for ~ 14 days and 1 μM methotrexate (MTX) for an additional 14 days. The CHO transfected pools were then evaluated and selected based on cell viability and gp120 expression (A244.AE or 6240.B). Proteins were purified from culture media of CHO transfected pools by lectin affinity chromatography and analyzed by RP-HPLC, SEC-HPLC and SDS-PAGE as previously described [44] for initial assessment of the quantity and integrity of the CHO expressed proteins.

Single CHO clones were then isolated from transfected cell pools by serial dilutions in 96-well plates. Positive clones were selected based on the cell viability and level of gp120 expression. The top ~ 20 clones for each construct were subjected to 12-week stability and 3 consistency cultures to select for the best-performing clones. In the stability study, a continuous parent culture was run for each clone with passaging every 4 days for 17 passages. The cells from the continuous culture were used to inoculate 3 intermittent terminal batch (TB) bioreactors, which were cultured for 7 days before harvest. Top CHO cell lines were selected based on the quantity and consistency of gp120 expression. The final cell lines for A244.AE or 6240.B gp120s were seeded at a density of 0.26 cells/mL in culture media and incubated at 36.5°C, 10% CO_2_ and shaken at 150 rpm for seven days. Cell cultures were harvested by centrifugation and the supernatants were sterile filtered prior to protein purification.

### Purification of A244.AE and 6240.B gp120 proteins from stable CHO cell line cultures

The harvested media were concentrated 5-fold and buffer exchanged into 20 mM Tris-HCl (pH 8.0 for A244.AE and pH 8.5 for 6240.B gp120) with 5 mM NaCl and loaded on a DEAE column (GE Life Sciences). The gp120 protein was eluted from the DEAE resin by 90 mM NaCl for A244.AE or 65 mM NaCl for 6240.B gp120. The eluted proteins were then buffer exchanged into 10 mM NaCl, 20 mM Tris-HCl (pH 8.0 for A244.AE and pH 8.5 for 6240.B gp120) and loaded onto a Fractogel SO3 (EMD) column. The Fractogel SO3 column captured additional contaminants and the purified gp120 product flowed through. The purified recombinant proteins were concentrated and buffer exchanged to 150 mM NaCl, 20 mM Tris-HCl pH 7.5 and finally, sterile filtered through a 0.22 μm membrane. The molecular weights and purity of the gp120 proteins were assessed by SEC-HPLC (Agilent SEC 3) and SDS-PAGE gel analysis. Throughout the purification process gp120 protein concentration was monitored by RP-HPLC. For this, media and intermediate purification products were injected on a Poros R1/10 column (PerSeptive Biosystems) equilibrated with 0.1% TFA in 5% ACN. gp120 proteins were eluted by a 30–35% ACN gradient in 0.1% TFA. gp120 elution peaks were selected and measured against standard gp120 A244.AE or 6240.B protein of known concentration. To evaluate the reproducibility of the protein production and purification processes, at least three independent batches were run and analyzed for each gp120 antigen.

For early analytical purposes, gp120s were purified using lectin affinity purification using agarose bound Galanthus nivalis lectin resin (GNA lectin; Vector Labs). The resin was initially washed 3 times with phosphate buffered saline pH 7.4 (PBS; Gibco) using 3 times the resin volume for each wash. Resin was resuspended at 50/50 (v/v) resin/PBS, and added directly to harvest culture media. 3 ml total resin was used per 100–150 ml cultures. The mixture was incubated at room temperature for 30 min with gentle rocking, and then poured over a gravity flow column (Bio-Rad), and media was allowed to flow through. Accumulated resin was washed with 5 column volumes PBS followed by gp120 elution using 3 column volumes of 1 M Methyl-alpha-D-Mannopyranoside added manually at approximately 1 ml/min. Eluted gp120 was dialyzed into 20 mM Tris pH 8.0, 150 mM NaCl buffer for analysis.

### Glycosylation analysis of the gp120 proteins

The gp120 proteins were denatured and reduced at 100°C for 10 min with RapiGest (Waters) and Dithiothreitol (DTT), at 0.1% (w/v) and 10 mM respectively, followed by alkylation with 20 mM Iodoacetamide (IAA) at room temperature for 30 min in the dark. Excess of IAA was quenched by adding DTT to a final concentration of 25 mM and incubated at room temperature for 15 min. The gp120 proteins were then de-N-glycosylated with PNGase F (New England Bio Labs) for 15 min, 60 min or overnight, or Endo H (New England Bio Labs) or Endo F3 (Sigma-Aldrich) for 8 hours at 37°C, as described by the manufacturer.

Deglycosylated proteins were digested with trypsin or pepsin using an enzyme/substrate ratio of 1/20 (w/w) and incubated overnight at 37°C. Prior to pepsin digestion, 1% (v/v) final concentration of formic acid (FA) was added (pH 2.0). Each digestion was subjected to an off-line desalting procedure using OASIS cartridges (Waters) following the manufacturer’s protocol and analyzed by LC-MS/MS on an ESI Synapt G2 or an ESI QTOF Premier mass spectrometer coupled to a NanoAcquity UPLC system (Waters). Samples were loaded onto a NanoAcquity 5 μm Symmetry C18 trapping column (180 μm x 20 mm, Waters), using full loop injection, for 3 min at a flow rate of 5 μl/min with mobile phase A (2% (v/v) ACN, 0.1% (v/v) FA) and were then separated on a NanoAcquity 1.7 μm BEH130 C18 analytical column (75 μm x 100 mm, Waters) using a 30-min gradient of 2–40% mobile phase B (98% (v/v) ACN, 0.1% (v/v) FA) at a flow rate of 300 nl/min.

Peptide identification was carried out from the generated peak list using the Mascot software 2.4 (Matrix Science, London, UK) run on a custom database comprising the sequences of the two recombinant gp120 proteins. The Mascot search parameters were set as follows: (a) enzyme: trypsin or unknown (for pepsin search); (b) missed cleavage: 1; (c) fixed modification: carbamidomethyl (C); (d) variable modifications: oxidation (M) and deamidation (N,Q) associated with HexNAc (N) or HexNAc(1)dHex(1) (N) or Hex(1)HexNAc(1) (T,S) or Hex(1)HexNAc(1)NeuAc(1) (T,S) or Hex(1)HexNAc(1)NeuAc(2) (T,S); (e) peptide tolerance 0.3 Da and (f) MS/MS tolerance of 0.3 Da. Glycopeptides assigned by Mascot search were confirmed by the MS/MS signal of characteristic sugar ions (*m/z* 204.1 for HexNAc, *m/z* 366.1 for HexNAcHex, *m/z* 292.1 for NeuAc, *m/z* 274.1 for NeuAc-H2O) and manually validated. The presence of sulfated residues was analyzed by using sulfation (S, T and Y) as variable modifications in the program MASCOT.

To assess the glycosylation pattern, the OASIS flow-through of each gp120 protein (A244.AE or 6240.B) treated with PNGase F and trypsin were completely dried in a Concentrator plus (Eppendorf) and resuspended with 10 μl of 0.1% TFA in 3% ACN. Samples (1 μl) were mixed on a ground steel target in a 1:1 ratio (vol/vol) with the matrix consisting of 2,5-DHB dissolved at 20 mg/ml in 0.1% (v/v) TFA in 30% (v/v) ACN supplemented with 1 mM NaCl and left to dry by air. The matrix crystals were made uniform by addition of 0.2 μl ethanol, causing rapid recrystallization.

Mass spectra were acquired on an MALDI-TOF/TOF Ultraflex mass spectrometer (Bruker Daltonics) in reflectron positive mode in the mass range of 700–3500 *m*/*z*. Spectra were externally calibrated by using a peptide calibration standard (Bruker Daltonics) spotted on the target. MS spectra were analyzed with FlexAnalysis 2.4 (Bruker Daltonics) using default parameters and manually revised. The obtained peak list was analyzed by GlycoWorkbench software 1.2.4105 a tool for the computer-assisted annotation of glycan mass spectra using in this case the internal database of CHO cells and literature match.

### Antigenicity measurements

Binding of the gp120 proteins to various analytes (sCD4 and mAbs) was determined using direct ELISA and Surface Plasmon Resonance (Biacore T200). For direct ELISA, either A244.AE or 6240.B gp120 protein was coated onto 96-well plates for 12 hours at room temperature. The wells were blocked with 1% (w/v) BSA in PBS for 60 min. Serial dilutions of human anti-gp120 IgG mAbs (with blocking buffer and 0.1% (w/v) Triton X-100 as diluent) were added to wells and incubated for 60 min, followed by a 3x rinse with PBS containing 0.05% Tween. For detection, a secondary anti-human Ab coupled with HRP (in diluent used above) was incubated for 60 minutes on the wells, followed by a 3x rinse with PBS containing 0.05% Tween. For development, TMB substrate (Rockland) was used, and the reaction stopped by adding 2.0 N sulfuric acid. All steps were carried out at room temperature. The optical density was determined spectrophotometrically at 450 nm wavelength using a microplate reader. EC50 values were calculated using Graphpad Prism software employing variable slope dose-response curves with a shared top and bottom to fit the data.

For the Biacore T200 (GE Healthcare, Uppsala, Sweden) antigenicity measurements, immobilization procedures were carried out using a standard amine coupling method with 10 mM Hepes, 150 mM NaCl, and 0.0025% Tween-20, pH 7.4 (HBS-0.0025%T). The CM5 chip surface was activated by a 1:1 mixture of 0.4 M 1-ethyl-3-(3-dimethylaminopropyl) carbodiimide hydrochloride (EDC) and 0.1 M N-hydroxysuccinimide (NHS) for 600 s with a flow rate of 10 μl/min. Next, 10 μg/ml of either anti-human IgG Fc (Bethanyl) or anti-RSV Synagis Palivizumab for a reference flow cell (MedImmune, Gaithersburg, MD) was immobilized to obtain 5,000–9,000 RU. The immobilized surface was then deactivated with 1.0 M ethanolamine-HCl pH 8.5 for 600 s. Different mAbs were captured (100–1,400 RU), followed by injection of different concentrations of A244.AE or 6240.B gp120 proteins. The bound surface was regenerated with 125 mM HCl for 45–60 s. Each kinetic assay was repeated at least three times. The data were analyzed using the Biacore T200 Evaluation software v1.0 (GE Healthcare, Uppsala, Sweden) and the data were fitted into a 1:1 Langmuir model using double subtraction (reference flow cell and the running buffer). Parameters including association rate constant (ka), dissociation rate constant (kd), maximum response (Rmax) and mass transfer constants (tc) were fitted globally. Closeness of the fit was assessed by chi-square (x^2^). For quality control, every kinetic assay required the following: mass transfer (t >10^8^), closeness of the fit (percentage of chi2/Rmax < 5%), and the uniqueness of the calculated rate constants and Rmax U value (< 25).

### Ethics statement

The animal study was conducted under an approved animal use protocol in an AAALAC accredited facility by the Institutional Animal Care and Use Committee at the former Novartis Vaccines organization (approval no. 09 NVD 044.3.3.09) in compliance with the Animal Welfare Act and in accordance with the principles set forth in the “Guide for the Care and Use of Laboratory Animals,” Institute of Laboratory Animals resources, National Research Council, National Academy Press, 2011 edition.

### Guinea pig immunizations

Female Hartley Guinea pig (Charles River Labs) with body weights of 250–275 g (10 animals per group) were immunized using bilateral intramuscular injections (250 μl (each) in the quadriceps on weeks 0, 4, 8, and 12 with 25 μg of each gp120 protein formulated with adjuvant MF59 (1:1 volume ratio) or adsorbed to aluminum hydroxide (AH, 3 mg/ml). These included Group 1 (6240.B + MF59), Group 2 (A244.AE + MF59), Group 3 (A244.AE + 6240.B + MF59), Group 4 (6240.B + AH), Group 5 (A244.AE + AH) and Group 6 (A244.AE + 6240.B + AH). Serum samples were collected prior to the first immunization (pre-bleed) and four weeks following the first, second, third and fourth immunizations (4wp1, 4wp2, 4wp3 and 4wp4, respectively). The method of anesthesia used during this study was Isoflurane and animals were monitored during treatment for respiratory rate, body temperature, heart rate and absence of pedal reflex. The method of sacrifice was C02 overdose with confirmation by exsanguination. Methods to reduce any pain or suffering during the study included the following procedures: Animals were monitored regularly for clinical signs and symptoms, at least once weekly from the time of receipt at facility; directly after the vaccination, then one day after the vaccination and then once a week, and during recovery from anesthesia, at one day post-procedure and weekly thereafter, and appropriate treatment was administered as required.

### Measurements of gp120- and epitope-specific binding Ab responses and virus neutralization assays

gp120- and scaffolded gp70-V1V2-specific binding Ab titers were measured by a standard ELISA format as previously described [44, 45]. Briefly, 0.1 μg/well A244.AE gp120, 6240.B gp120, gp70-V1V2 AE.92TH023 or gp70-V1V2 B.CaseA2 proteins were coated on 96-well plates at 4°C overnight. Coated gp70-V1V2 proteins were blocked with 0.5% milk in 1x PBS, 0.1% Tween 20, pH 7.4 (blocking and diluent buffer) while coated gp120 proteins were blocked with superblock blocking buffer (Pierce). Next, sera were serially diluted in the diluent buffer described above. In each case, plates were washed in PBS + 0.1% Tween 20. Upon washing, complexes of serum Abs with gp70-V1V2 and gp120 proteins were incubated with 1:10,000 and 1:30,000 dilutions of HRP-conjugated goat anti-guinea pig IgG (Southern Biotech), respectively. The coated plates were then incubated with Peroxidase Substrate (KPL, Inc.), followed by phosphoric acid addition to stop the reaction, and absorbance was measured at 450 nm wavelength.

Binding Abs reactive with cyclic V2 and V3 peptides were measured using N-terminal biotinylated peptides (JPT Peptides, Berlin, Germany) using SPR Biacore 4000 (GE Healthcare, Uppsala, Sweden). The surface of sensor chip CM5 was activated using EDC/NHS amine coupling followed by immobilization of 1 μM Streptavidin (Life Tech, USA) in 10 mM sodium acetate pH 4.5 to obtain 4,000–5,000 RU. The immobilized surface was then deactivated with 1.0 M ethanolamine-HCl pH 8.5 for 600 s. Spot 3 in each flow cell was left unmodified to serve as a reference. After the surface deactivation, the following cyclic biotinylated peptides were captured to obtain 1,200–2,200 RU:

V2.AE (92TH023): CSFNMTTELRDKKQKVHALFYKLDIVPIEDNTSSSEYRLINC
V3.AE (92TH023): CTRPSNNTRTSINIGPGQVFYRTGDIIGDIRKAYC
V2 (6240.B): CSFNITTGIGNKMQKEYALFYKLDVVPIDSNNNSDNTSYRLISC
V3 (6240.B): CTRPNNNTRKGIHIGLGQALYATGDIIGDIRQAH

For SPR measurements evaluating gp120-specific binding Abs in guinea pig immune sera, the A244.AE or 6240.B gp120 proteins were immobilized directly on the activated EDC/NHS CM5 chip to obtain 250–5,000 RU. Following the surface preparation, control mAbs and individual guinea pig serum samples were diluted (1:300) in 10 mM HEPES, 300 mM NaCl and 0.005% Tween 20. Quadruplicate samples were injected at a flow rate of 10 μl/min at 25°C onto the protein captured surface for 250 s followed by a dissociation period of 1800 s. The signal was then enhanced with a 200 s injection of 30 μg/ml goat anti-guinea pig IgG Ab. To regenerate the bound surface, 125–250 mM HCl was injected for 60 s and repeated twice. Data analysis was performed using Biacore 4000 Evaluation Software 4.1 (GE Healthcare, Uppsala, Sweden). The binding Ab responses were then normalized using double subtraction to the unmodified reference surface and the blank buffer injection). For avidity analysis, the 1:1 (Langmuir) dissociation fitting was applied, given by the formula, R0 * exp(-kd * (t—t0)) + Offset, where kd is the dissociation rate constant (1/s); R0 is the response units at the start of the fitted data; t0 is time at the start of the fitted data; and offset is the residual response units at infinite time.

Linear epitope mapping was performed as previously described [46, 47] with some modifications. Briefly, array slides were provided by JPT Peptide Technologies GmbH (Berlin, Germany) by printing a peptide library designed by Dr. B. Korber (Los Alamos National Laboratory) onto Epoxy glass slides (PolyAn GmbH, Germany). The library contains overlapping peptides (15-mers overlapping by 12) covering 7 full-length HIV-1 gp160 Env consensus sequences (Clades A, B, C, D, Group M, CRF1 and CRF2 [19]) and six vaccine strain gp120 sequences (MN.B, 1086.C, TV1.C, ZM651.C, A244.AE, and 92TH023.AE) [47]. All serum samples were diluted 1:50 and hybridized to the slides using a Tecan HS4000 Hybridization Workstation, followed by incubation with DyLight 649-conjugated goat anti-Guinea Pig IgG (Jackson ImmunoResearch, West Grove, PA). Fluorescence intensity was measured using a GenePix 4300 scanner (Molecular Devices, Sunnyvale, CA) and analyzed with GenePix software. Binding intensity of the post-immunization serum to each peptide was corrected with its own background value, which was defined as the median signal intensity of the pre-bleed serum for that peptide plus 3 times the standard error among the 3 sub-arrays on the slide.

Virus neutralizing Ab activity was quantified using the well-standardized assay employing HIV Tier 1 and 2 pseudoviruses and a luciferase reporter gene assay in TZM-bl cells as reported previously [48].

### Statistical methods

Graphical distributions of response magnitudes are plotted for each group and time point with box plots superimposed on the distributions magnitudes. The mid-line of the box denotes the median and the ends of the box denote the 25^th^ and 75^th^ percentiles. The whiskers denote the most extreme data points that are no more than 1.5 times the interquartile range (i.e., height of the box). As necessary, the plots are drawn on the base 10 log scale. Only significant p values less than 0.05 are displayed above the boxplots when comparing two groups. To determine a difference in response magnitude between groups at specific time points the Wilcoxon rank-sum test was used based on the exact distribution and using the mid-ranks method for ties [49]. To determine a difference in response magnitudes between time points the Wilcoxon signed-rank test was used, using the exact method and the Pratt method for handling zeros [50]. All tests are two-sided and considered significant at alpha of 0.05. Due to the exploratory nature of this analysis multiple comparison adjustments were not made here. R version 3.3.1 was used to create all boxplots and the coin package was used for testing.

## Results

### Generation of stable CHO cell lines and methods of production of HIV-1 subtypes CRF-01 AE (A244.AE) or B (6240.B) gp120 protein

Following a consultation with HIV vaccine experts assembled by the US Military HIV Research Program and the National Institute of Allergy and Infectious Diseases in November, 2011, recombinant gp120s from the HIV-1 subtypes CRF_01 AE A244 (A244.AE) and B 6240 (6240.B) were selected as potential candidate protein boosts for consideration for future clinical trials in Thailand [40]. The A244.AE and 6240.B gp120 coding sequences with 11 amino acids deleted from N-terminus (Δ11 gp120) were used for the generation of stable CHO cell lines, and initial evaluations were performed to assess the expression levels and integrity of the gp120s.

**Fig 1:**
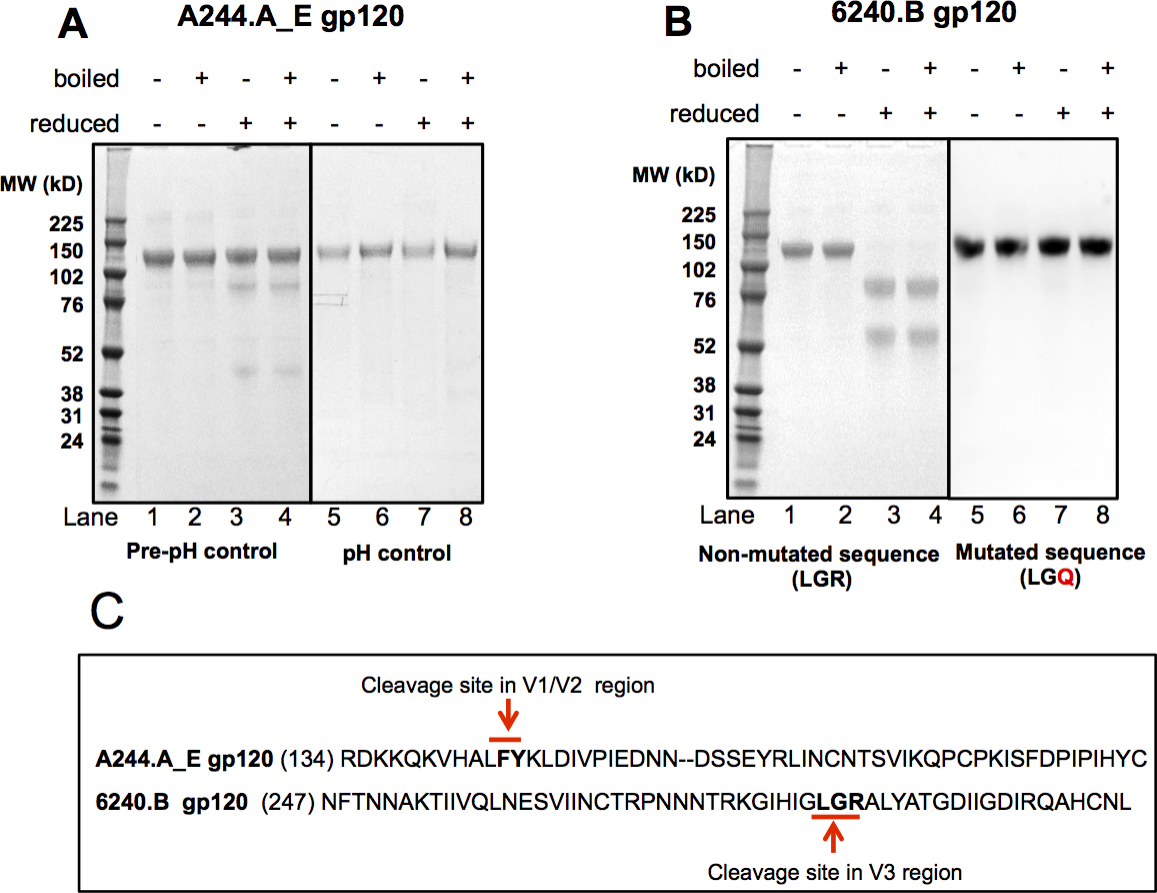
Purification of uncleaved A244.AE and 6240.B gp120 proteins. (**A**) SDS-PAGE of A244.AE gp120 before (left panel) and after (right panel) pH control during gp120 production. The ladder and the gp120 separation were run on the same gel. However, the gel included a lane that was unrelated to this study so the ladder is displayed separately on the image. In addition, on the right panel, there was a pen mark on one lane and it was kept as an original image. (**B**) SDS-PAGE of 6240.B gp120 before (left panel) and after (right panel) site mutation, a cleavage site within the V3 loop. (**C**) Amino acid sequences corresponding to protease sensitive regions of d11 A244.AE and 6240.B gp120s. The identified protease cleavage sites are designated by arrows.

Initial SDS-PAGE evaluation of A244.AE gp120 purified from the culture media of transfected CHO cell pools showed a single gp120 band under non-reducing conditions (**Fig 1A,** lanes 1, 2), and three bands, one dominant intact gp120 band and two cleaved gp120 bands by SDS-PAGE under reduced conditions (**Fig 1A,** lanes 3, 4). N-terminal sequencing (performed at Tufts University Core Facility, Boston, MA) of the cleaved bands showed that the cleavage site was in the V2 loop of gp120 and consensus to that for aspartic acid protease (**Fig 1C**), which is known to be active at acidic pH. To inhibit the aspartic acid protease activity, the A244.AE gp120 was purified from the CHO cell culture media while maintaining a neutral pH (pH 7.0 +/- 0.2) resulting in the purification of intact protein without any cleavage products (**Fig 1A,** lanes 5–8).

Similarly, SDS-PAGE analysis of the 6240.B gp120 transfected CHO cell supernatants showed one intact gp120 band under non-reducing conditions (**Fig 1B** lanes 1, 2), but no or very little intact gp120 and two smaller MW bands under reduced conditions (**Fig 1B**, lanes 3,4), indicating that the 6240.B gp120 protein was also subjected to protease cleavage. N-terminal sequencing of the cleaved bands showed that the cleavage site was in the V3 loop of gp120 and consensus to a trypsin-like protease cleavage site (**Fig 1C**). In contrast to the A244.AE gp120 situation, pH control during purification did not appear to prevent this cleavage effectively with preparations of this molecule. Therefore, to prevent this cleavage, a variant gp120 6240.B construct was generated with a single site mutation of Arg to Gln at p1’ at the cleavage site, and transfected into CHO cells to generate CHO cell pools for screening. The gp120 purified from these transfected pools of cells appeared as a single intact gp120 band under both reducing and non-reducing conditions with no cleaved bands detectable (**Fig 1B**, lanes 5–8). This confirmed that this single site mutation in the V3 region completely prevented cleavage of 6240.B gp120.

To derive clonal CHO stable cell lines expressing A244.AE and 6240.B gp120s, single cells were isolated from the transfected CHO cell pools and evaluated for expression levels, integrity of the product, and stability as described in Methods. The final top clones expressing each of the A244.AE and 6240.B gp120 antigens were identified that consistently produced yields of > 200 mg/L in batch cell cultures. Once top cell lines were established, methods to consistently generate intact gp120 proteins with high purity and high yields were developed with features that would be suitable for future clinical product manufacturing. For several reasons (related to raw material availability, potential effects on glycan composition, residual lectin, etc.), lectin affinity purification was not considered for this purpose. The platform that was developed included two chromatography steps and three ultrafiltration/diafiltration steps (UF/DF). DEAE anion exchange chromatography captured gp120 protein while contaminants either flowed through or strongly bound to the column. The target gp120 product was eluted from the column with increased NaCl concentration. Fractogel SO3 cation exchange further separated gp120 monomer from aggregated proteins, with gp120 monomer flowing through and gp120 aggregates and other contaminants remaining bound to the column. The purification platform was applied for each of the A244.AE and 6240.B gp120s with buffer components optimized separately for each. With this platform, each of the A244.AE and 6240.B gp120 proteins were produced as monomeric gp120 with high purity (>95% as measured by SEC-HPLC) and with minimal or no detectable cleavage products following three independent production/purification (“consistency”) runs (**S1 Fig**).

### Glycosylation analysis of the CHO cell line produced gp120s

Analyses of the glycosylation of gp120 molecules have become an important aspect of HIV-1 Env vaccine development [51, 52], and have received increased attention since it has been shown that glycans contribute to epitopes recognized by several broadly neutralizing and potent mAbs [53, 54]. The N-linked and O-linked glycans of the A244.AE and 6240.B gp120 proteins were analyzed using a combination of enzymatic deglycosylation and proteolytic digestion coupled with LC-MS/MS.

**Fig 2:**
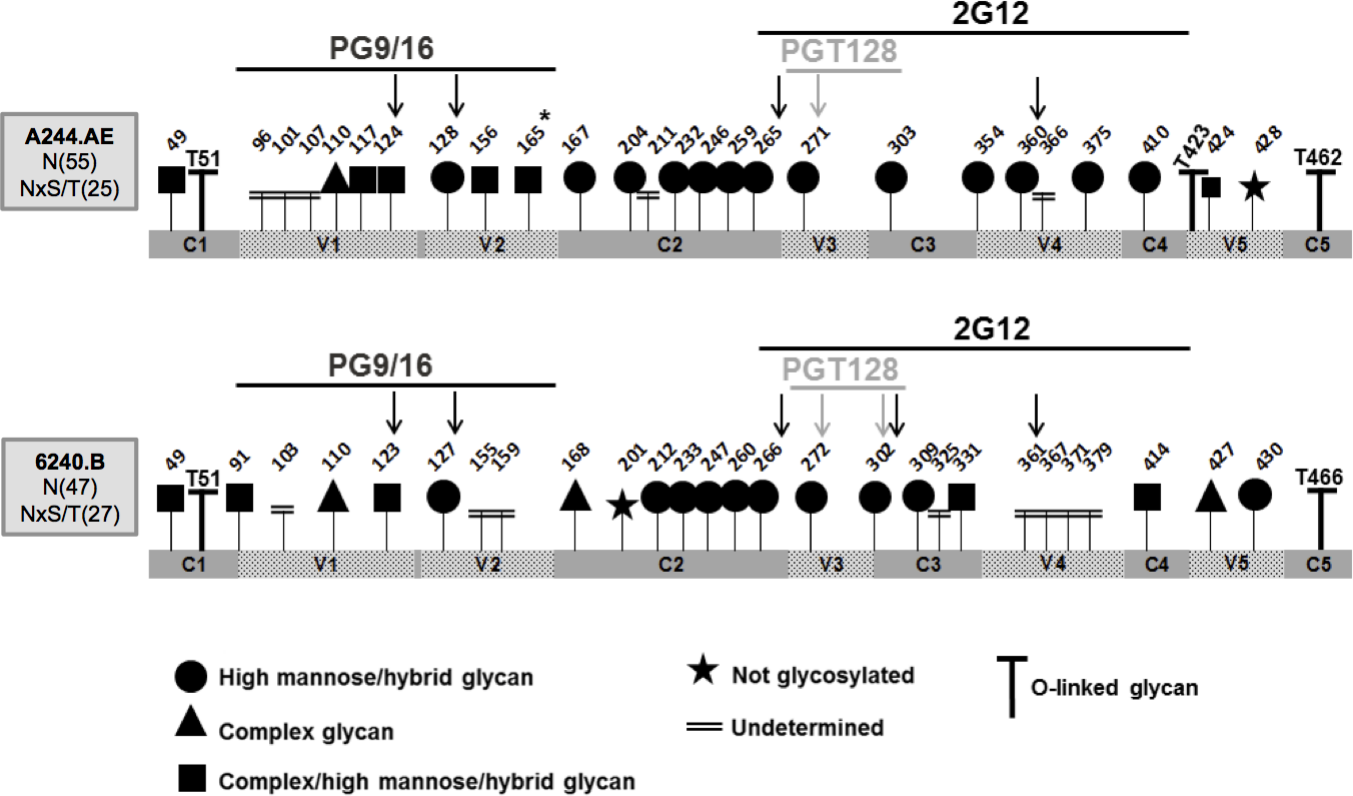
N-linked and O-linked glycosylation of the A244.AE and 6240.B gp120 proteins. N-linked and O-linked glycosylation sites of the A244.AE gp120 (upper panel) and 6240.B gp120 (lower panel) were identified by LC-MS/MS analysis after enzymatic deglycosylations (PNGase F, Endo H or Endo F3) and proteolytic digestions (trypsin or pepsin) of the proteins. The identified N-linked glycosylation sites are represented by the following: circles correspond to high mannose/hybrid glycans; triangles, to complex glycans; squares, to complex high mannose/hybrid glycans; double underscore, undetermined glycan type (asparagine residues identified as aspartic acid residues after treatment with PNGase F). Non-glycosylated asparagine residues in a consensus motif are reported with a star. Glycosylated asparagine residues in a non-consensus motif (N165 of A244.AE) is indicated with an asterisk. The “T” in the figure indicates the O-linked glycosylations identified for both proteins. The figure also reports the variable loops (V1-V5), conserved sequences (C1-C5) and the glycan epitopes mapped by PG9/16, 2G12 and PGT128 mAbs.

Initially, the potential N-linked glycosylation sites (PNGS), which fit with the N-linked glycosylation consensus motif (N-X-S/T, X being any amino acid but Pro) were assigned when an asparagine residue was identified as an aspartic acid residue after treatment with PNGase F. Because sample treatment can allow the deamination of asparagine to aspartic acid and lead to a misinterpretation of the N-linked glycosylation sites, the glycoprotein gp120s were also treated with PNGase F for a short period followed by pepsin digestion. To differentiate glycosylated sites with complex or high mannose/hybrid glycans, the glycoprotein gp120s were deglycosylated with Endo H that catalyzes the cleavage of high mannose/hybrid glycans and Endo F3, which catalyzes the cleavage of complex glycans. These two glycosidases cleave N-glycans leaving only one GlcNAc intact on the asparagine residues and the corresponding modification could be easily detected by LC-MS/MS. The N-consensus glycosylation sites on both A244.AE and 6240.B gp120s were mapped to the five variable loops (V1-V5) and the five constant regions (C1-C5). Of the twenty-five A244.AE gp120 PNGS, only the N428 was found unmodified (**Fig 2**). Thirteen PNGS were exclusively modified by high mannose/hybrid glycans (substrate of PNGase and Endo H), one was exclusively modified by complex glycans (substrate of PNGase and Endo F3), six were found modified by complex and high mannose/hybrid glycans (substrates of PNGase, Endo H and Endo F3), and at last, undetermined glycans were assigned to five PNGS (substrates of PNGase). It is noteworthy that the N165 of the A244.AE gp120, a non-consensus glycan motif in the V2 region was found to carry either complex or high mannose/hybrid glycan. The modification was identified from the fragmentation of the tryptic peptide ^163^LINCNTSVIKQPCPK^177^ (**Fig 2** and **S1 Table**). From the twenty-seven 6240.B gp120 PNGS, only the N201 was found unmodified (**Fig 2** and **S2 Table**). Ten PNGS were exclusively modified by high mannose/hybrid glycans, three were exclusively modified by complex glycans, five were found modified by both complex and high mannose/hybrid glycans and undetermined glycans were assigned to eight PNGS.

In contrast to N-linked glycan sites, O-glycosylation sites have not yet known to be associated with specific consensus sequences. For this reason, the study of these glycosylations remained an analytical challenge. The strategy that we followed was to identify O-linked glycosylation sites by treating the molecules with PNGase F, Endo H or Endo F3 and trypsin coupled with LC-MS/MS. Those peptides bearing a serine or threonine modified with Hex(1)HexNAc(1), Hex(1)HexNAc(1)NeuAc(1) or Hex(1)HexNAc(1)NeuAc(2) lead to a mass difference of +365, +656 or +947 Da, respectively, from the non-modified amino acids residues. Three peptides derived from A244.AE gp120 containing T51, T423 and T462 and two peptides derived from 6240.B gp120 containing T51 and T466 were identified as O-glycosylated. While O-glycosylation near the N-terminal (T51) and C-terminal of both gp120 molecules have been previously reported [55], this study revealed a new O-glycosylation site T423 in V5 domain of A244.AE gp120. The corresponding site on 6240.B gp120 was also found to be a threonine residue but a corresponding glycopeptide was not identified. Based on the accurate mass, all the O-glycans were predicted to have core 1 mono-or di-sialylated GalNAc-Gal structures. All the identified glycopeptides are provided in supplementary data (**S1** and **S2 Tables).**

Finally, the glycan fractions obtained from PNGaseF and trypsin treatment of both A244.AE and 6240.B gp120 proteins were analyzed by MALDI-TOF/TOF MS. The two mass spectra, from each of the two treated gp120 glycoproteins, were analyzed by Glycoworkbench3 software for the computer-assisted annotation of glycan mass spectra using, in this case, an internal database of predicted glycan structures for CHO cells as well as information available in the literature [56, 57]. The comparison of the mass spectra revealed a similar and heterogeneous glycosylation profile mainly composed of high mannose and complex glycans consistent with what has been previously described for other CHO-cell-produced gp120s including the MN and A244 gp120 products used in the AIDSVAX B/E vaccine boost of the RV144 trial [51, 58].

### Antigenicity of the gp120 proteins

**Fig 3:**
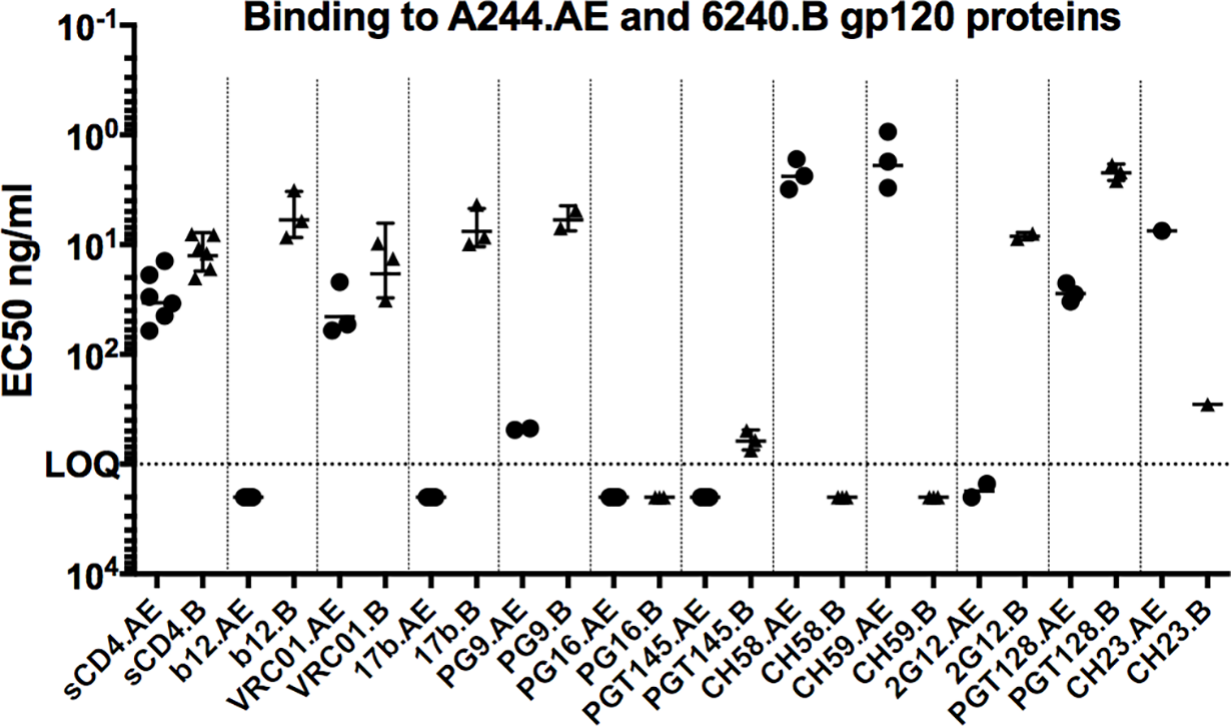
ELISA-EC50 analysis of A244.AE and 6240.B gp120 binding to mAbs recognizing critical epitopes. ELISA-EC50 values for the binding of the gp120s to a panel of well-characterized mAbs were determined as described in Methods. All results shown here were confirmed by Surface Plasmon Resonance (SPR; some confirmatory analyses and additional data were shown for an expanded panel of mAbs in S4 Fig and S5 Fig. EC50s of mAb binding to A244.AE gp120 (circles) and binding to 6240.B gp120 (triangles) were shown. Each symbol (circle/triangle) represents a replicate measurement.

Two independent assay platforms, ELISA EC50 and Surface Plasmon Resonance (SPR), were used to analyze the binding of the purified gp120s to a panel of well-characterized mAbs, b12, VRC01, 17b, CH58, CH59, PG9, PG16, 2G12, PGT128, PGT145, and CH23 and soluble CD4 (sCD4). Results from this initial analysis using ELISA showed that the A244.AE gp120 bound sCD4 [59], VRC01 (recognizing CD4 binding site (CD4bs) [60], CH58 and CH59 (recognizing a linear epitope in V2) [43], PGT128 (recognizing two conserved glycans and short β-strand segment of the gp120 V3 loop) [61], and CH23 (linear epitope in the V3 crown) [62–64]. It showed a low level of binding to PG9 (recognizing V2 apex), b12 (CD4bs) [65], and no measurable binding to PGT145 (binding to HIV-1 Env trimer apex via a long ß-hairpin HCDR3) [66, 67], CD4bs (b12 [68] and 17b [69]), PG16 [70] and 2G12 (recognizing N-linked carbohydrates in the C2, C3, V4, and C4 regions of gp120) [71, 72] (**Fig 3**). In the ELISA assay, the 6240.B gp120 bound sCD4 as well as mAbs against CD4bs epitopes (b12, VRC01), the CD4 inducible (CD4i) region (17b), V2 apex (PG9), a glycan-specific epitope (2G12) and a V3-glycan-specific mAb (PGT128). 6240.B gp120 did not bind Abs directed against a V2 linear peptide epitope in CRF01_AE (CH58, CH59), which had been also previously shown by Liao *et al*., [43] nor V2 apex mAb, PG16. Binding to PGT145 and CH23 was at the lower limit of detection (**Fig 3**).

To further explore the kinetics of mAb binding to the A244.AE and 6240.B gp120 proteins, SPR analyses were performed using the CD4bs mAbs, VRC01 and VRC03, and an expanded panel of mAbs that binds to the conformation-dependent V2i epitope that targets the integrin binding site in V2 (mAbs 697-30D, 1393A, 830A and 2158) [73] and the V3 crown (mAbs 2219 and 2557) (**Table 1, S3** and **S4 Figs**). For mAbs tested by both SPR and ELISA (**Fig 3**) the data for each mAb are consistent. It was noteworthy that the expanded data from SPR showed that two of the four mAbs that target V2i, mAbs 697 and 1393, had different patterns with A244.AE and 6240.B in that both mAbs had slower on and off rates with the former versus the latter (kd values of 5.25 nM and 0.53 nM for mAbs 697-30D and 1393A versus A244.AE and kd values of 0.02 nM and 0.02 nM for mAbs 697-30D and 1393A versus 6240.B, respectively (**Table 1** and **S3I, S3J, S3M and S3N Fig**). As a control for non-specific binding, mAbs 1418, 2F5 and 4E10 were also employed.

**Table 1:** Antigenicity analyses of antigens and mAbs targeted to CD4bs, V1V2, V2 and V3 regions.

mAbs	ka/kd/KD	A244.AE gp120	6240.B gp120
	ka (x 10^3^ M-1 s-1)	9.95 ± 0.947	25.4 ± 1.28
VRC01 (CD4 bs)	kd (10^−3^ s-1)	0.549 ± 0.0044	1.32 ± 0.004
	KD (nM)	**55.5 ± 6.75**	**52.2 ± 1.32**
VRC03 (CD4 bs)	KD (nM)	N/A	N/A
	ka (x 10^3^ M-1 s-1)	N/A	1.18 ± 0.118
PG9 (V1V2)	kd (10^−3^ s-1)	N/A	0.601 ± 0.0304
	KD (nM)	**N/A**	**51.8 ± 6.92**
PG16 (V1V2)	KD (nM)	N/A	N/A
	ka (x 10^3^ M-1 s-1)	91.8 ± 2.83	
CH58 (V2)	kd (10^−3^ s-1)	0.144 ± 0.00793	
	KD (nM)	**1.58 ± 0.123**	N/A
	ka (x 10^3^ M-1 s-1)	52 ± 1.42	
CH59 (V2)	kd (10^−3^ s-1)	0.0468 ± 0.00914	
	KD (nM)	**0.904 ± 0.20**	N/A
	ka (x 10^3^ M-1 s-1)	40 ± 1.66	31.8 ± 1.80
697-30D (V2)	kd (10^−3^ s-1)	5.25 ± 0.0449	0.0208 ± 0.000267
	KD (nM)	**131 ± 5.52**	**0.656 ± 0.0428**
	ka (x 10^3^ M-1 s-1)	11.1 ± 3.08	42.3 ± 0.28
1393A (V2)	kd (10^−3^ s-1)	0.53 ± 0.0734	0.0155 ± 0.000297
	KD (nM)	**48.7 ± 5.69**	**0.367 ± 0.00814**
	ka (x 10^3^ M-1 s-1)	60.9 ± 0.58	24 ± 0.75
830A (V2)	kd (10^−3^ s-1)	0.0482 ± 0.000147	0.0478 ± 0.000235
	KD (nM)	**0.791 ± 0.00721**	**0.199 ± 0.00132**
	ka (x 10^3^ M-1 s-1)	37.2 ± 0.45	48.6 ± 0.56
2158 (V2)	kd (10^−3^ s-1)	0.055 ± 0.00125	0.0191 ± 0.000883
	KD (nM)	**1.48 ± 0.0471**	**0.393 ± 0.0224**
	ka (x 10^3^ M-1 s-1)	55.7 ± 9.36	144 ± 25
2219 (V3)	kd (10^−3^ s-1)	5.01 ± 0.868	37.6 ± 29.1
	KD (nM)	**90.1 ± 9.24**	**243 ± 163**
	ka (x 10^3^ M-1 s-1)	178 ± 1.51	354 ± 9.99
2257 (V3)	kd (10^−3^ s-1)	5.71 ± 0.18	1.42 ± 0.00116
	KD (nM)	**32 ± 1.11**	**4.01 ± 0.146**

Although no broadly neutralizing Ab activity to the V2 apex was observed in RV144 vaccinees, Abs directed to the V2 region were an important correlate of reduced risk of infection in the RV144 vaccine trial [15–17]. We therefore looked further at the binding of the two gp120s using a comprehensive panel of V2-specific mAbs recognizing the “V2p” linear epitopes (CH58, CH59), conformational V2 apex epitopes (PG9 and PG16), and the “V2i” mAbs that recognize a putative α4β7 integrin binding motif at ^179^LDI/V^181^, distal to the PG9 and CH58 epitopes; these include the V2i mAbs 697-30D) [74], 830A, [75], 1393 [73, 76], and 2158 [74]. V2 apex mAb PG9 did not bind to A244.AE gp120, but did bind to 6240.B gp120 (KD: 51.80 nM) and PG16 did not bind either A244.AE or 6240.B gp120 (**Table 1** and **S3 Fig**). Both CH58 and CH59 mAbs bound to A244.AE gp120 protein with high affinity; with a KD of 1.58 nM for CH58, and 0.90 nM for CH59, while no binding was observed with the 6240.B gp120 protein. As noted above, V2i mAbs 697-30D and 1393A bound to both the A244.AE gp120 and to 6240.B gp120s but with much less affinity than did V2i mAbs 830A and 2158. All four of these V2i mAbs however showed higher binding to the 6240.B gp120 as compared to the binding seen with the A244.AE gp120. (**Table 1** and **S3 Fig**). Overall, the high binding affinity was observed between all four tested V2i mAbs to both A244.AE and 6240.B antigens. Comparison binding affinity of V2i mAbs to A244.AE and 6240.B antigens, the binding of all four V2i mAbs to 6240.B exhibited higher binding affinity than that of the A244.AE antigen (**Table 1**). The highest binding affinity on all tested mAbs was binding between V2i 830A mAb and 6240.B antigen (KD = 0.199 nM). Moreover, all V2i mAbs bound to A244.AE and 6240.B with fast on and slow off rates.

The V3-specific mAb 2219 (recognizing an epitope in beta-hairpin of the V3 crown), [77, 78] was shown to bind both the A244.AE and 6240.B gp120 proteins with KD of 90 nM and 243 nM, respectively (**Table 1** and **S4 Fig**). Higher affinities were observed with the V3 crown mAb 2557 [79] for both A244.AE (32 nM) and 6240.B (4.01 nM) proteins. In contrast to the V2i mAbs, the V3 mAbs showed relatively slow on and fast off rates. The two CD4bs mAbs VRC01 and VRC03 showed different binding properties with VRC01 exhibiting similar KD for A244.AE (55.5 nM) and 6240.B (52,2 nM) proteins while VRC03 [80] bound neither A244.AE or 6240.B gp120 (**Table 1** and **S4 Fig**).

### Immunogenicity of the gp120 proteins in guinea pigs

**Fig 4:**
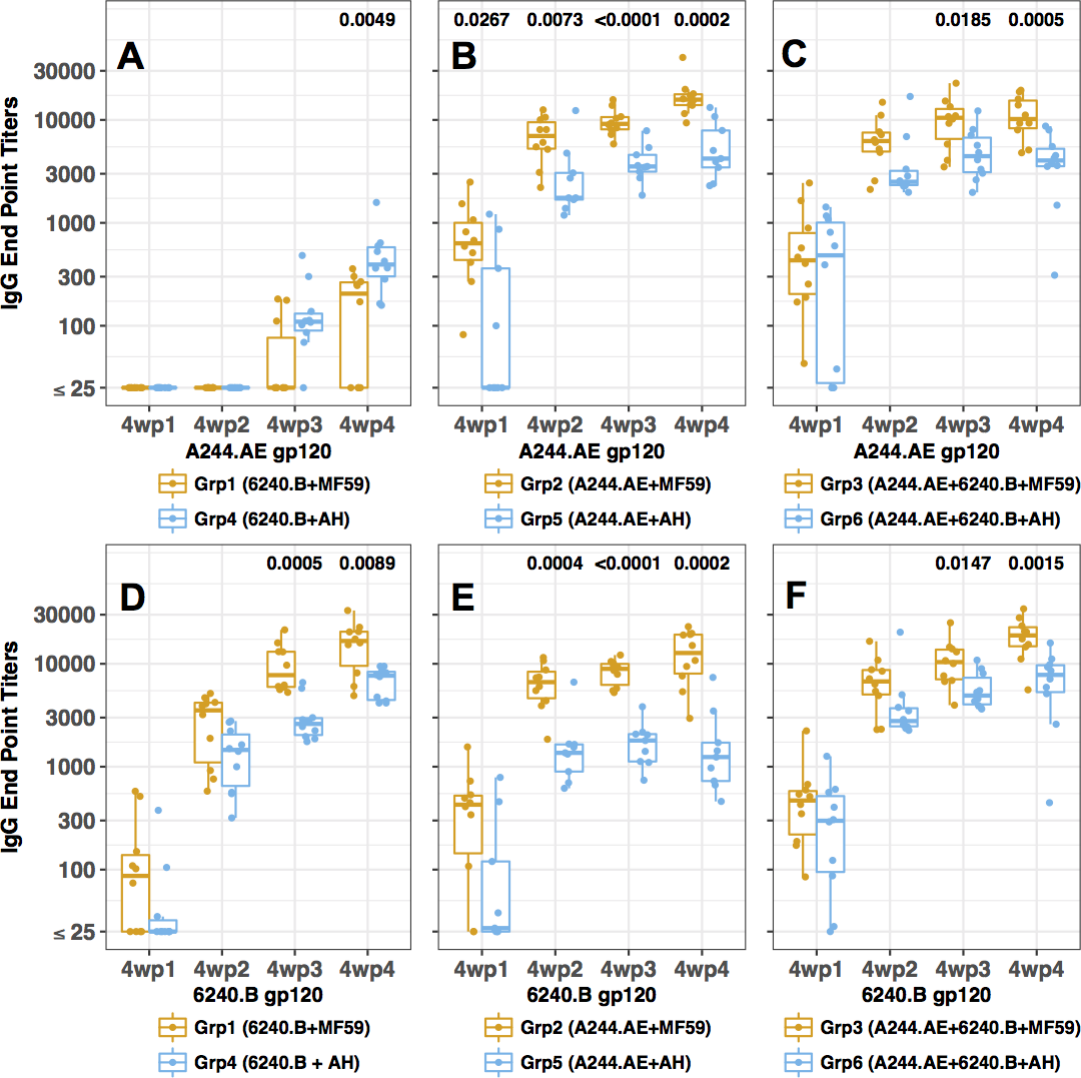
gp120-specific Ab responses in guinea pigs following immunization with A244.AE and 6240.B gp120s. Six groups of ten guinea pigs each were immunized with monovalent gp120 vaccine formulations [Grp1 (6240.B + MF59), Grp4 (6240.B + AH), Grp2 (A244.AE + MF59), Grp5 (A244.AE + AH)], or bivalent gp120 vaccines {Grp3 (A244.AE + 6240.B + MF59), Grp6 (A244.AE + 6240.B + AH)} as described in Methods. Sera were conducted at four weeks following each immunization (4wp1, 4wp2, 4wp3 and 4wp4). (**A—C**) A244.AE gp120 protein was coated onto the 96-well plates. (**D—F**) 6240.B gp120 protein was coated onto the 96-well plates. Each symbol represents the end-point-titer for an individual animal with geometric mean titers indicated for each animal within a group. Differences between groups (MF59 vs. AH) were compared using Wilcoxon rank-sum test with confidence level of p < 0.05.

**Fig 5:**
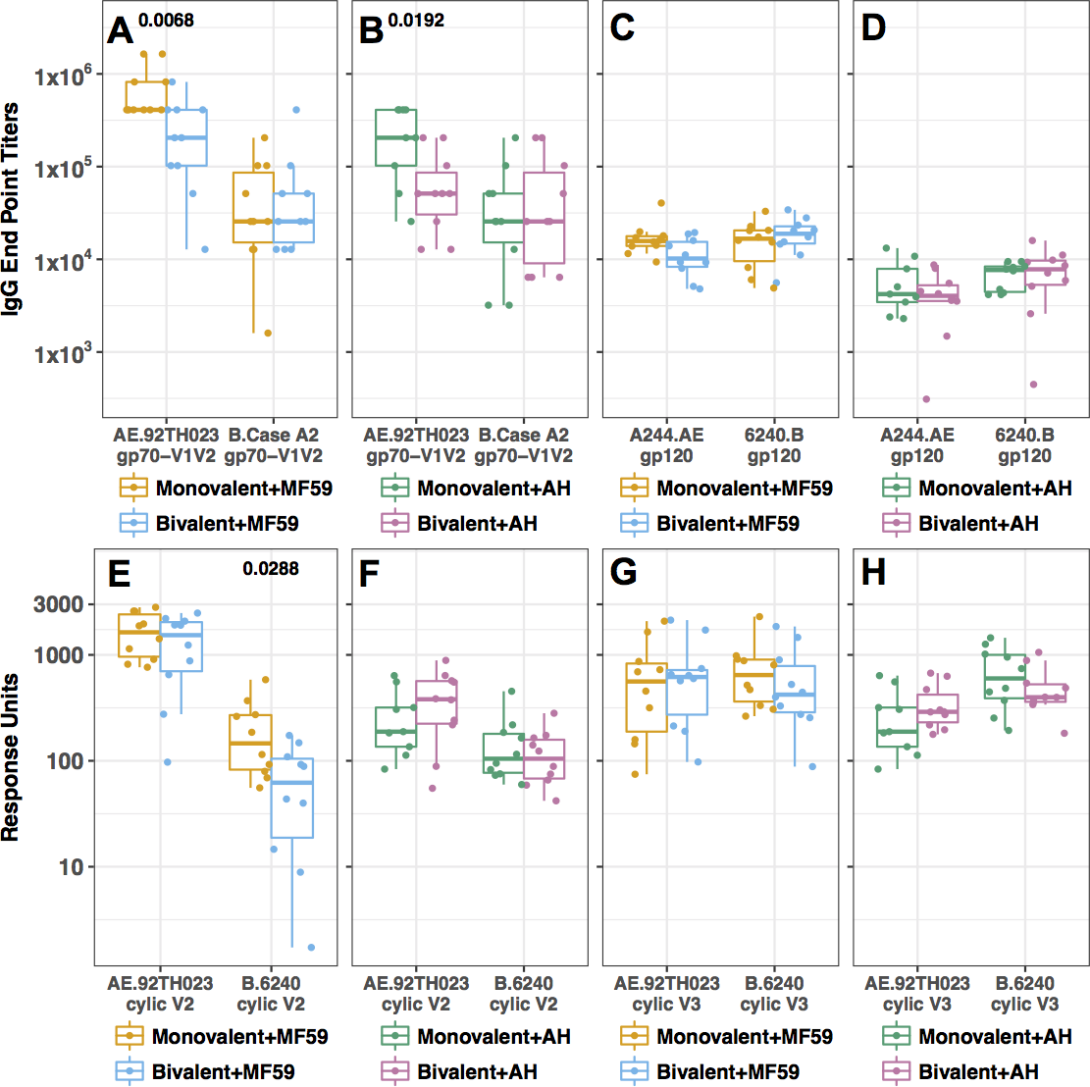
Comparison of Ab responses immunized with monovalent and bivalent gp120s formulated in AH and MF59 adjuvants. (**A**) Ab responses to subtype- matched gp70-V1V2 proteins in MF59 groups. (**B**) Ab responses to subtype-matched gp70-V1V2 protein in AH groups. (**C**) Ab responses to strain/subtype-matched gp120s in MF59 groups. (**D**) Ab responses to matched gp120 to AH groups. (**E**) Ab responses to V2 cyclic peptides in MF59 groups. (**F**) Ab responses to V2 cyclic peptides in AH groups. (**G**) Ab responses for V3 cyclic peptide in MF59 groups. (**H**) Ab responses for V3 cyclic peptide in AH groups. Differences between groups (monovalent vs. bivalent) were compared using Wilcoxon rank-sum test with confidence level of p < 0.05 with significant differences indicated by corresponding p values above relevant groups. Monovalent designation is meant to indicate the immunization group receiving the monovalent gp120 in each panel that matches the subtype of the reagent used to measure binding.

To evaluate the immunogenicity of the two gp120 proteins, groups of ten female Hartley guinea pigs were immunized with 25 μg of each of the A244.AE (Grps 2 and 5) or 6240.B gp120 proteins (Grps 1 and 4), or a bivalent combination (Grps 3 and 6; 25 μg for each protein). The gp120s were formulated with either a proprietary oil-in-water emulsion MF59 (Grps 1–3) or AH (Grps 4–6) adjuvant. Overall, robust gp120 binding Ab responses to one or both protein antigens were observed following vaccination in all groups with significant titers measurable after the second immunization and peak titers observed following the 4^th^ immunization (**Fig 4)**. Interestingly, the MF59-adjuvanted gp120 proteins showed significantly higher gp120-specific IgG titers against matched or mismatched gp120 proteins than that of the AH-adjuvanted vaccines groups (**Fig 4B–4F)** except in the case of cross-subtype binding of immunized 6240.B proteins (Grp 1 or Grp4) to A244.AE protein antigen (**Fig 4A**). Animals immunized with the 6240.B gp120 protein with MF59 or AH showed high titer binding Ab responses against the strain/subtype-matched 6240.B gp120 protein (**Fig 4D)**, but low titers against the strain/subtype-mismatched A244.AE gp120 (**Fig 4A**). In contrast, animals immunized with the A244.AE gp120 with MF59 showed robust and nearly comparable binding Ab responses against the matched and mismatched gp120 antigens (**Fig 4B** and **4E**). A244.AE gp120 AH immunized animals showed lower titers overall and more appreciably reduced titers against the mismatched antigens (**Fig 4B** and **4E**). Compared to monovalent and bivalent immunization groups, the induction of Abs to either A244.AE or 6240.B gp120 proteins was comparable **(Fig 4** and **Fig 5C and 5D).**

### Antibody responses to the variable loop regions of the HIV-1 Env

**Fig 6:**
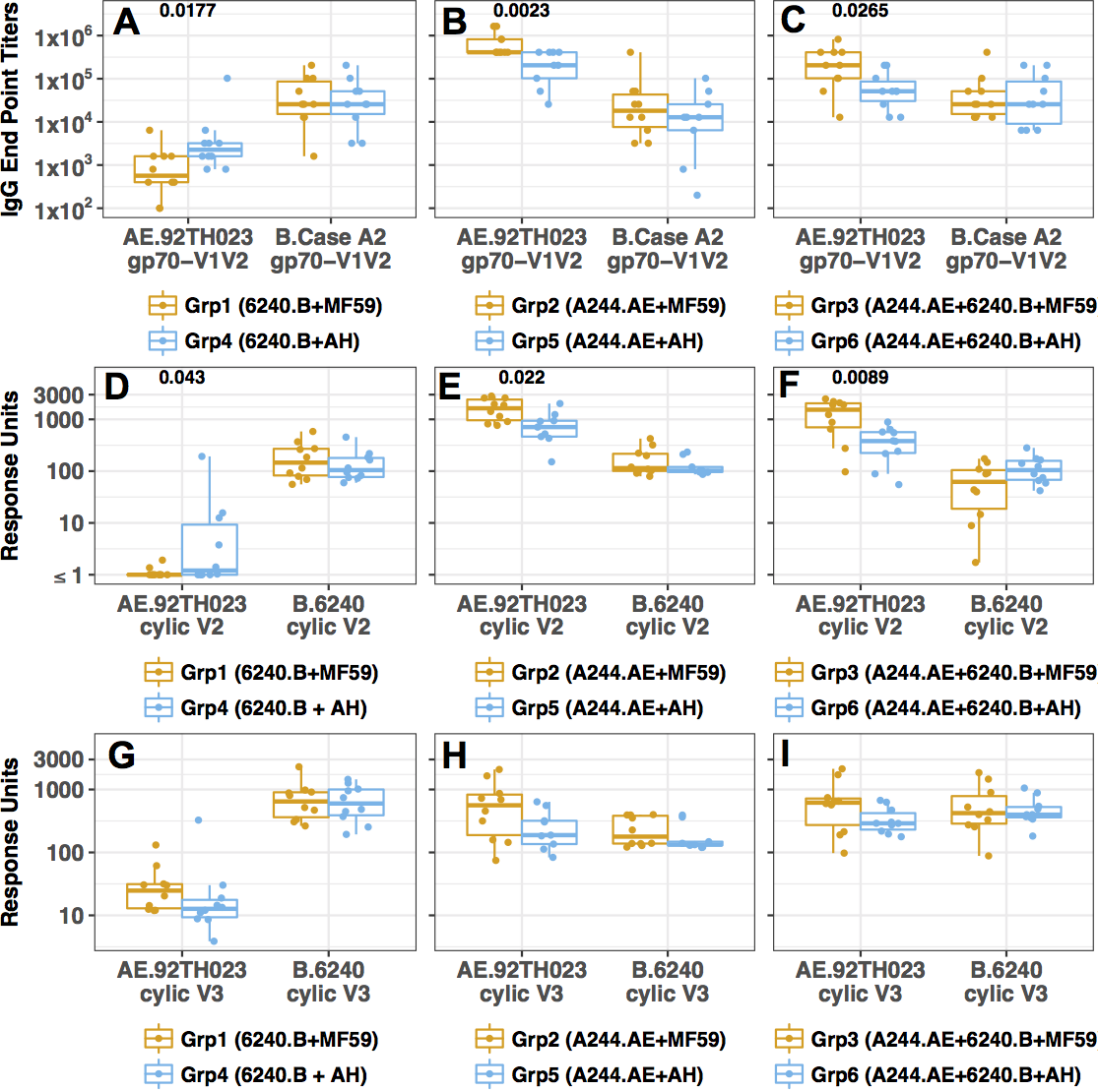
gp70-V1V2 protein and cyclic V2- and V3-peptide-specific Ab responses in guinea pigs. **(A-C)** Ab responses to gp70-V1V2 AE.92TH023 and gp70-V1V2 B.Case A2 proteins were evaluated by ELISA. Ab responses for the cyclic V2 (**D-F**) and V3 peptides (**G-I**) were measured by SPR. In each panel, comparisons of epitope-specific Ab responses elicited in animals immunized with vaccines formulated with MF59 or AH are shown: **(A, D, G)** monovalent 6240.B (Grp 1 vs. Grp4); **(B, E, H)** A244.AE (Grp2 vs. Grp5) and **(C, F, I)** bivalent (Grp3 vs. Grp6). **(A-C)** Ab responses to gp70-V1V2 by ELISA; (**D-F**) Ab responses to cyclic V2 peptides by SPR; (**G-I**) Ab responses to cyclic V3 peptides by SPR. Each symbol for the ELISA represents the mean end-point titer for an individual animal analyzed in triplicate. The SPR assays were conducted with 4 replicates and are represented as the response units. The values shown in each panel represent the geometric mean response units for each group. Differences between groups (MF59 vs. AH) were compared using Wilcoxon rank-sum test with confidence level of p < 0.05. The p values are provided above groups that showed statistically significant differences.

Further evaluations of epitope-specific binding Abs were performed using guinea pig sera collected following the fourth immunization when responses against gp120 were at their highest (**Fig 4**). Binding Ab responses to gp70-scaffolded V1V2 proteins were measured using ELISA, and responses to cyclic V2 and V3 peptides were analyzed using SPR. While cross-clade Ab binding activity was elicited by each gp120, the responses were clade-biased in that gp120 6240.B gave higher responses to gp70 antigens scaffolded with gp70-V1V2 B.CaseA2 rather than gp70-V1V2 AE.92TH023, and *vice versa*. Sera from groups immunized with 6240.B gp120 with MF59 and AH (Grp1 and Grp4) showed similar high binding Ab titers to the gp70-V1V2 B.CaseA2 protein **(Fig 6A).** These animals also elicited Ab responses that cross-reacted with the gp70-V1V2 AE.92TH023, but the magnitude was much lower than that measured with the gp70-V1V2 B.CaseA2 protein, and the AH group showed higher cross-reactive titers than the MF59 group (**Fig 6A**; p = 0.0177). Sera from animals immunized with the A244.AE gp120 protein (Grp2 and Grp5) showed high titer responses against the gp70-V1V2 AE.92TH023 and substantial cross-reactivity to the gp70-V1V2 B.CaseA2 (**Fig 6B**). Sera from the A244.AE gp120 MF59 group showed significantly higher Ab titers to gp70-V1V2 AE.92TH023 as compared to those from the AH group (**Fig 6B**, p = 0.0023), consistent with the higher gp120-specific Ab responses measured in the MF59 group (**Fig 4**). Immune sera from animals receiving the bivalent gp120 formulations (Grps 3 and 6) showed substantial Ab responses to each of the scaffolded V1V2 proteins as was seen with the monovalent A244.A_E gp120 groups (**Fig 6B and 6C** and **Fig 5A and 5B)**, and again, the Ab responses to the gp70-V1V2 AE.92TH023 were significantly higher in the bivalent MF59 group (p = 0.0068) as compared to the AH group (p = 0.0192). Nevertheless, sera from both groups given bivalent vaccine with either MF59 or AH, surprisingly, showed significant reductions in their binding Ab titers against gp70-V1V2 AE.92TH023 as compared to those seen with the A244.E gp120 monovalent groups with the same adjuvants, respectively **(Fig 5A and 5B;** p = 0.0068, 0.0192, respectively). In contrast, they showed similar Ab titers against the gp70-V1V2 B.CaseA2 compared to either monovalent group **(Fig 5A and 5B)**. These observations should be further explored in dose ranging studies with these gp120 antigens administered alone and in in combination.

Ab responses directed to epitopes within the V2 and V3 regions of Env were evaluated by SPR using full-length cyclic peptides corresponding to these regions. The Ab responses against the cyclic peptides were generally poorly cross-reactive and far more biased than the responses measured with the gp70-V1V2 antigens. For the subtype CRF01_AE, V2 and V3 peptides from the 92TH023 strains were used, while for subtype B, a polypeptide with the matched sequences from 6420.B HIV- strain was employed (**S5 Fig)**. The 6240.B gp120 vaccinated animals showed modest binding Ab responses to the matched 6240.B cyclic V2 peptide and no or low cross-reactivity to the AE.92TH023 V2 peptide (**Fig 6D**). The groups immunized with the A244.AE gp120 protein elicited 10-fold higher binding to their subtype-matched AE.92TH023 cyclic peptide than did the 6240.B gp120 groups to their sequence-matched cyclic V2 peptide, and also showed some low level cross-reactivity with the subtype B cyclic V2 peptide (**Fig 6E**). The A244.AE gp120 MF59 group showed 2-fold higher Ab binding responses to AE.92TH023 cyclic V2 peptide as compared to AH group (**Fig 6E**, p = 0.022). In contrast to what was observed with gp70-V1V2 AE.92TH023 peptide (**Fig 5A and 5B**), similar responses to the AE.92TH023 cyclic V2 peptide were obtained following immunization with the bivalent or monovalent formulations (**Fig 5E and 5F**). However, the bivalent gp120 MF59 group appeared to show a reduced response to the 6240.B cyclic V2 peptide as compared to the responses elicited by the 6240.B gp120 monovalent vaccine **(Fig 5E,** p = 0.0288).

The 6240.B gp120 protein elicited Ab binding responses to the 6240.B cyclic V3 peptide in both the MF59 and AH immunization groups although at very low levels (**Fig 6G**). Compared with the observed Ab binding responses against the 6240.B cyclic V2 peptide, Grp1 (6240.B gp120 MF59) and Grp4 (6240.B gp120 AH) showed 3.7- and 4.7-fold higher responses to the cyclic V3 peptide, respectively (**Fig 6G vs. 6D**). In contrast, immunizations with the A244.AE gp120 protein induced 2.4-fold and 3.7-fold lower Ab responses to V3 as compared to those against V2 for Grp2 (MF59) and Grp5 (AH), respectively (**Fig 6H vs. 6E**). However, the MF59 group (Grp2) still induced 2.4-fold higher V3 Ab binding response than Grp5 with AH (**Fig 6H**), similar to the 2.1-fold higher V2 Ab binding responses (**Fig 6E**). Immunization with the bivalent protein formulations elicited overall similar Ab binding responses to the two cyclic V3 peptides tested, but the MF59 group (Grp3) showed slightly higher responses (2.1 fold) against the AE.92TH023 V3 than the AH group (Grp6) (**Fig 6I**).

### Antibody responses to linear epitopes in Env

**Fig 7:**
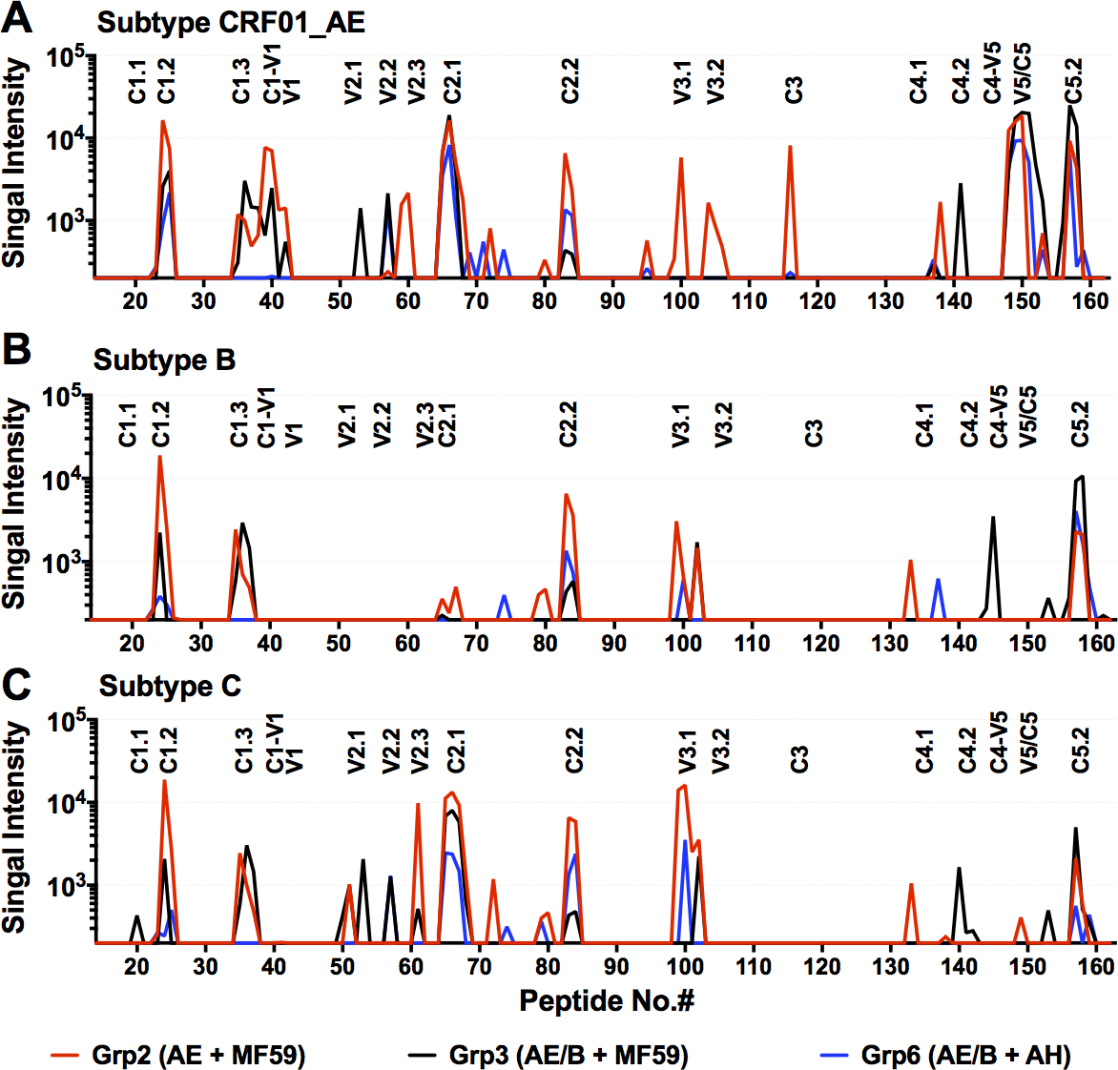
Linear epitope mapping of Ab responses in guinea pig immune sera. Immune sera from 5 guinea pigs showing the highest Ab responses from groups 2 (A244.AE + MF59), 3 (A244.AE + 6240.B + MF59), and 6 (A244.AE + 6240.B + AH) were used to map Abs to the linear epitopes spanning the entire gp120 sequence. The signal intensities for the median of five animals for each epitope positions were plotted for the three groups. Panels **A, B, and C** show serum Ab reactivity to the subtypes CRF01_AE, subtype B, and subtype C peptides, respectively.

Ab responses to linear epitopes spanning the entire gp120 molecule were mapped using the peptide microarray assay format [47]. The linear peptide sets included sequences of the HIV-1 subtype CRF01 AE A244, and TH023 strains, subtype B MN, and subtype C 1086, TV1, and ZM651 strains, as well as the consensus Env sequences for subtypes A, B, C, D, CRF01-AE, CRF02-AG, and group M [19]. For this analysis, guinea pig sera from five out of ten animals at 4 weeks post-fourth immunization in groups 2, 3 and 6 were evaluated. The highest magnitude of linear epitope-specific Ab responses to the linear peptide sequences from subtypes CRF01_AE, B, and C were seen in the animals immunized with the A244.AE gp120 in MF59 (Grp2) followed by the bivalent gp120 MF59 group (Grp3) then the bivalent gp120 AH group (Grp6) (**Fig 7, S7 Fig** and **S3 Table**). Groups 2, 3, and 6 all developed Abs that targeted linear epitopes in C1, V2, C2, V3, C3, C4, V5, and C5 regions of gp120, with the magnitude and clade preference varying among groups. Comparison of CRF01_AE-binding profiles revealed comparable binding response to CRF01_AE C1.2, C1.3, C1/V1, C2.1, C2.2, V5/C5 and C5.2 epitopes (**Fig 7** and **S3 Table**). The bivalent Group 3 (MF59) induced significantly Ab responses to C5.2 epitope compared to bivalent group 6 (AH) (p = 0.0317 for subtype CRF01_AE and B, **S3 Table**). The monovalent A244.AE gp120 MF59 vaccine (Grp2) induced binding Ab responses against V2.3, V3.1, V3.2, C3 and C4.1 epitopes that were distinguishable from other groups, while the bivalent MF59 vaccine (Grp3) elicited binding Ab responses to V2.1, V2.2 and C4.2 epitopes distinguishable from other groups. Specifically, binding to the CRF01 AE V2.1 epitope was significantly higher (p = 0.0317, **S3 Table**) for the bivalent MF59 group (Grp3) compared to the A244.AE M59 group (Grp2), and binding to the clade C V2.1 epitope was significantly higher for bivalent MF59 group when compared either to the A244.AE M59 group (p = 0.0397, **S3 Table**) or the bivalent AH group (p = 0.0317, **S3 Table**). In addition, the bivalent MF59 (Grp3) generated Abs binding to subtype B C4/V5 peptides while other groups did not. Interestingly, the Abs generated in all four groups cross-recognized the subtype C peptides showing a similar pattern of reactivity as compared to that seen with the CRF01_AE peptides (**Fig 7** and **S7 Fig**). The bivalent MF59 animals (Grp3) showed a unique pattern of reactivity to V2.1 and V2.2, the most critical regions associated with decreased HIV-1 risk in RV144 [19]. Statistical analysis of the Ab reactivity to the individual epitope regions showed significant differences between the monovalent and bivalent MF59 groups; however the total signal intensity remained the highest levels in the Group 2 (A244.AE gp120 + MF59) (**S7 Fig and S3 Table**). Overall, the bivalent MF59 animals (Grp3) induced Abs targeted the broadest epitopes and the most important epitopes of V2.1, which has been shown in the HIV-1 vaccine efficacy study. Moreover, these vaccine formulations induced Abs cross-reactive multiple HIV-1 subtypes (CRF01-AE, clade A, B,C, D, and CRF02-AG) tested in this study, manifesting considerable breadth of the binding Ab response.

### gp120-specific antibody binding avidity in guinea pig immune sera

**Fig 8:**
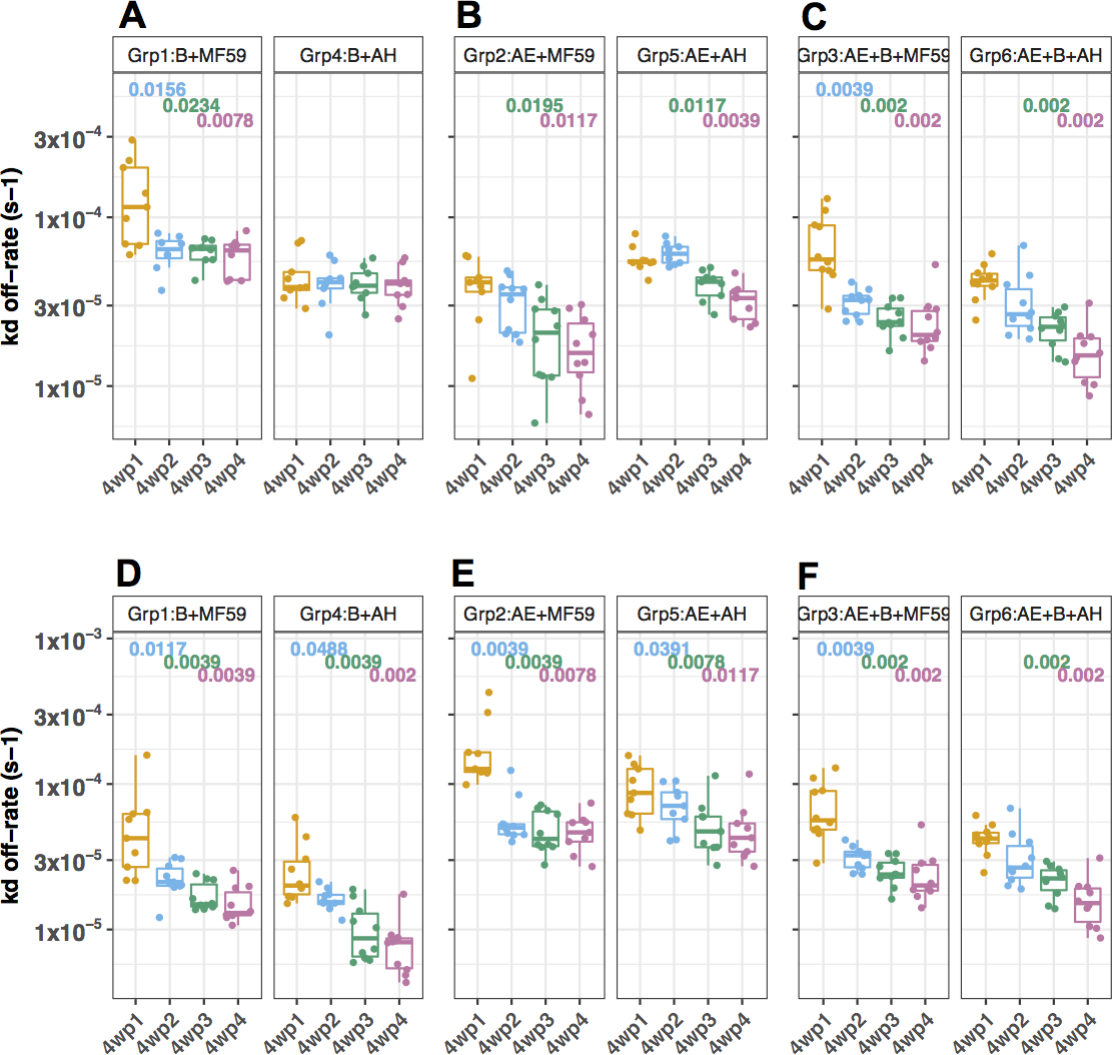
Evaluation of the avidities (kd off-rate) of Abs-specific to A244.AE and 6240.B gp120s by SPR. Either A244.AE (A, B, C) or 6240.B (D, E, F) gp120 protein was immobilized on CM5 chip and the diluted sera were injected onto the immobilized CM5 chip followed by extended dissociation– 30 min. The SPR assay was conducted using different immobilized densities with more than 3 replicates and arithmetic mean was used for each group. The dissociation fitting was conducted using 1:1 binding model. Avidity for A244.AE gp120 for (**A**) monovalent Grp1 and Grp4. (**B**) monovalent Grp2 and Grp5. (**C**) bivalent Grp3 and Grp6 and Avidity for to 6240.B gp120 for (**D**) monovalent Grp1 and Grp4. (**E**) monovalent Grp2 and Grp5. (**F**) bivalent Grp3 and Grp6. Differences between groups (MF59 vs. AH) were compared using Wilcoxon rank-sum test with confidence level of p < 0.05. The p-values indicated show comparisons of 4wp1 vs. 4wp2, 4wp1 vs. 4wp3, 4wp1 vs. 4wp4 within the same group. Multiple comparisons were shown in the S4 and S5 Tables.

The avidity of Ab binding to the gp120 proteins (as dissociation constants, kd) was measured in guinea pig immune sera collected at 4-week intervals over the course of the study by SPR using either A244.AE or 6240.B gp120 protein immobilized onto sensor chips as described in the Methods section. When the A244.AE gp120 protein was used, gp120-specific Abs reached higher avidity with statistical significance after each sequential immunization in all groups formulated with the MF59 adjuvant (**Fig 8A–8C**, **S4** and **S5 Tables**). Increased Ab avidities were also measured over the course of immunization in the groups receiving AH formulated vaccines. Ab avidities following the final immunizations (4wp4, 4^th^) were significantly higher in the MF59 groups as compared to the AH groups for A244.AE gp120 in the comparison of Grp2 vs. Grp5 at 4wp4 (p = 0.0023) (**Fig 8B** and **S4 Table**). In case of the comparison for the bivalent groups between MF59 and AH, both groups induced high avidity binding Abs directed to either of the A244.AE and 6240.B gp120 proteins; however, the bivalent group (Grp6) of AH induced higher avidity than Grp3 of MF59 at 4wp4 (p = 0.0433, for binding to either of the gp120s (**Fig 8C and 8F** and **S4 Table**). Moreover, no statistically significant differences were observed between monovalent and bivalent groups using either MF59 or AH for A244.AE gp120 protein (**S6A Fig**) while monovalent group induced significantly higher avidity than bivalent groups for 6240.B gp120 protein (**S6B Fig**). When similar SPR analyses were performed with the 6240.B gp120 protein on the sensor chip, increased Ab avidities were also seen following sequential immunizations (**Fig 8D–8F**, **S4** and **S5 Tables**). As was seen with the A244.AE gp120 specific Ab responses, all groups rapidly elicited high Ab avidities specific to the subtype 6240.B gp120 protein (**Fig 8D–8F**, **S4** and **S5 Tables**). In conclusion, both MF59 and AH groups showed high Ab avidities that were achieved over the period of repeated immunizations and likely reflect the maturation of Abs in the lymph node germinal centers by somatic hypermutation.

### Evaluation of virus neutralizing antibody responses

As a measure of the functionality of the Ab responses to the gp120 proteins, virus neutralization assays were performed with sera collected at baseline and after the fourth immunization using the standardized TZM.bl assay (**S8 Fig**). Guinea pigs immunized with the A244.AE gp120 developed robust neutralizing activity against the Tier 1a CRF01_AE TH023.6 pseudovirus (**S8A Fig, middle panel**). Moderate neutralizing activity against the Tier 1a subtype B MN.3 and SF162. LS strains (**S8B and S8C Fig, middle panel**), and strong cross-subtype neutralizing activity against the Tier 1a subtype C MW965.26 pseudovirus (**S8D Fig, middle panel**) were also measured. Moreover, significantly higher neutralizing Ab titers were measured in animals that received the monovalent A244.AE gp120 in MF59 as compared to A244.AE gp120 AH for TH023.6, MN.3, SF162.LS, and MW965.26 (**S8A–S8D Fig**, p = 0.0431, p = 0.0325, p = 0.0216, p = 0.0101, respectively). The groups immunized with the bivalent gp120 formulations showed similar virus neutralizing Ab profiles as those immunized with the monovalent A244.AE gp120 alone. In contrast, sera from animals immunized with 6240.B gp120 in either MF59 or AH showed no or very low neutralizing Ab responses against both the Tier 1a subtype B strains (MN.3 and SF162. LS, (**S8B and S8C Fig**); and only low titers of neutralizing Abs were seen against Tier 1a subtype C pseudovirus, MW965.26 (**S8D Fig, left panel**).

## Discussion

The results of the RV144 Phase III HIV vaccine trial provided a rationale and key insights into the design of future prime boost regimens in Thailand, as well as in other key populations in the world, which are hardest hit by HIV/AIDS. To conduct such trials, it is important to evaluate whether the Env subunit protein vaccines selected for the essential boost component, as well as for the antigen(s) used for the vector prime, can elicit responses against the HIV strains relevant to the regions where the vaccines are tested in order to provide optimal coverage. Therefore, for future post-RV144 clinical trials in key populations at risk for HIV-1 subtypes CRF01_AE and B, two gp120 candidates were evaluated from the subtypes B 6420 and CRF01_AE A244 HIV-1 strains, and stable CHO cell lines and methods were developed to produce these vaccine materials.

To achieve the required high level, stable expression of homogeneous intact gp120 molecules for potential future manufacturing, the antigens were first subjected to modifications that included codon-optimization and N-terminal truncation. Subsequently, a two-step ion-exchange purification method was developed that could be used for both candidates that offered the purity, integrity, consistency and high yields necessary for cost effective vaccine production with the potential to be broadly applicable to diverse HIV gp120s. Early analysis of purified gp120 6240.B and A244.AE revealed substantial clipping in the V3 and V1/V2 regions, respectively. Elimination of the proteolytic clipping with a single amino acid substitution in the V3 region, in the case of gp120 6240.B, and with pH control during the purification process in the case of A244.AE allowed for the production of intact and homogeneous products with good recoveries.

In addition, the complex glycosylation pattern of the HIV Env has highlighted the importance of characterizing these residues in vaccine preparations [58]. Therefore, both immunogens gp120 A244.AE and 6240.B were extensively evaluated for both N-linked and O-linked glycosylation patterns. Using a combination of enzymatic deglycosylation and mass spectrometry analysis, N- and O-linked glycans were mapped along the five variable loops and five constant regions of the A244.AE and 6240.B gp120 candidates. Twenty four of the twenty five A244.AE PNGS and twenty six of the twenty seven 6240.B PNGS were found at least partially glycosylated, with high mannose glycan clusters around C2-V3-C3-V4-C4 domains as previously reported for other gp120s [51]. Most importantly some of these represent broadly neutralizing mAb binding domains [61, 81, 82]. Moreover, we confirmed for both proteins, the presence of O-glycosylation sites near N- and C-terminal of the primary sequences [26, 55, 83]. Emerging from this analysis is the unusual observation that a residue within the V2 region of A244.AE, N165, is modified by either a complex or high mannose/hybrid glycan despite falling within a motif not usually glycosylated in eukaryotes. The N165 is part of the unusual sequons NXN and NXQ that have been only reported in very few cases for glycoproteins of eukaryotic origin [84, 85]. To the best of our knowledge this type of glycosylation has so far never been reported in viral glycoproteins. In addition, this study revealed a new O-glycosylation site, T423, in V5 domain of A244.AE gp120. The impact of these unique modifications is not yet known but their description is key as part of a full structural understanding of the molecules. Lastly, although not extensively investigated here, a posthoc analysis of the data generated in this study did not reveal the presence of sulfated tyrosines in V2 or in any other segments of the two proteins as has been suggested by other studies.

The purified A244.AE and 6240.B gp120 proteins also were shown to bind with high affinities to an overlapping and complementary set of HIV Env-specific ligands including sCD4 as well as mAbs recognizing the CD4 binding site (CD4bs) epitopes, the CD4 inducible (CD4i) region, and V1/V2 and V3-epitopes, some of which include conserved glycan residues known to play a critical role in virus neutralization. Importantly, the results shown here for the A244.AE gp120 prepared from the new stable CHO lines with new purification methods were the same as those seen with purified preparations representative of the HSVgD-tagged A244 gp120 used in RV144, and were consistent with those previously published using other preparations of the A244 gp120 antigen [40].

Immunization of guinea pigs with either the monovalent or bivalent gp120 proteins formulated in either the MF59 or AH adjuvants was shown to elicit high titers of gp120-binding Abs against the vaccine antigens, and in the case of the A244.A-E gp120, high titers of Abs cross-reactive with the mismatched subtype B 6420.B gp120. In addition, all vaccine preparations elicited high level Ab responses to at least one of the gp-70V1V2 reagents tested, an important observation since binding to the gp70-V1V2 B.CaseA2 protein, in particular, was shown to be inversely correlated with the rate of HIV infection in RV144 [15, 17]. In our study, Ab responses to the gp70-V1V2 B.CaseA2 of subtype B following immunization of guinea pigs was in general lower than that seen against the gp70-V1V2 AE.92TH023 reagent. This might be in part because the lower degree of amino acid sequence identity between the gp70-V1V2 B.CaseA2 and the 6240.B gp120 as compared to that between the gp70-V1V2 AE.92TH023 and A244.AE. However, A244AE gp120 immunized animals showed substantial titers of Abs that were cross-reactive with the clade mismatched gp70-V1V2 B.CaseA2 protein. In RV144, vaccinated subjects with higher plasma IgG targeting linear V2 and V3 also showed lower rate of HIV-1 infection [19]. Animals immunized with A244.AE gp120 induced higher Ab binding titers to the clade-matched AE cyclic V2 peptide than those seen with animals immunized with the 6240.B gp120 protein against their clade and sequence matched V2 peptide. The 6240.B gp120 groups showed greater reactivity with clade-matched cyclic peptide.

Importantly, the peptide array analysis of immune sera showed that only the bivalent vaccine formation with MF59 (Grp3) showed a unique pattern of reactivity to V2.1 and V2.2, the most critical regions associated with protection in RV144 [19]. This provided further support to the use of multivalent vaccine preparations with more potent adjuvants such as MF59 as well as others in future clinical trials with these and similar vaccines. Indeed, results recently emerging from the HVTN100 Phase 1 clinical trial showed that a bivalent subtype C gp120 vaccine boost formulated with MF59 elicited robust Ab and T-cell immune responses at levels that met the criteria for advancement to proof of concept efficacy testing in the HVTN702 Phase IIb/III trial in the southern African region [29]. The MF59 adjuvant used here represents one of the few adjuvants approved for human use having been first approved for the influenza vaccine, FLUAD^TM^, in Italy in 1997, and more recently, in the USA in 2015 [86].

To further build upon this approach of using more potent adjuvants in post-RV144 clinical trials, additional Phase I studies will test other promising adjuvants such as AS01 shown to be clinically safe and effective in the setting of malaria vaccination [87]. AS01 is a liposome-based vaccine adjuvant system containing two immune stimulants: 3-*O*-desacyl-4ʹ-monophosphoryl lipid A (MPL) and the saponin QS-21 [88]. With the combination of the MPL and QS-21, vaccines using the AS01 adjuvant have been shown to elicit strong antigen-specific polyfunctional CD4+ T-cell responses against multiple pathogens including varicella-zoster [89] and hepatitis B [90]. Moreover, different formulations of MPL with or without QS-21 have been proposed for various vaccine antigens including HIV-1 Env [91, 92]. The gp120 immunogens developed here should be further tested with safe and potent adjuvant formulations such as these to further evaluate their preclinical immunogenicity and protective efficacy and potential utility for future clinical studies.

## Supporting information

S1 Fig Demonstration of the consistency of A244.AE and 6240.B g120 production and purification processes. SDS-PAGE of purified **(A)** A244.AE gp120 protein and (**B**) 6240.B gp120 protein from three independent rounds of production and purification, respectively. It was noticed that there were several pen marks on some lanes and they were kept with an original image.
(TIF)

S2 Fig Mass spectra obtained from the MALDI-TOF/TOF analysis of the two gp120 glycan fractions. (TIF)

S3 Fig SPR analysis of gp120 binding to mAbs specific to V1V2 loops or V2 loop. Anti-human IgG Fc was immobilized on the sensor CM5 chip followed by mAb V1V2/V2 capture. Next, various concentrations of A244.AE/6240.B gp120 protein were injected onto the captured mAb V1V2/V2 surface. The sensograms were shown in color codes for different concentrations of gp120 proteins. The kinetics values shown in **S1 Table** were calculated on an average from at least three replicates. (**A**) mAb PG9 vs. A244.AE gp120; (**B**) mAb PG9 vs. 6240.B gp120; (**C**) mAb PG16 vs. A244.AE gp120; (**D**) mAb PG16 vs. 6240.B gp120; (**E**) mAb CH58 vs. A244.AE gp120; (**F**) mAb CH58 vs. 6240.B gp120; (**G**) mAb CH59 vs. A244.AE gp120; (**H**) mAb CH59 vs. 6240.B gp120; (**I**) mAb 697 vs. A244.AE gp120; (**J**) mAb 697 vs. 6240.B gp120; (**K**) mAb 830A vs. A244.AE gp120; (**L**) mAb 830A vs. 6240.B gp120; (**M**) mAb 1393 vs. A244.AE gp120; (**N**) mAb 2158 vs. 6240.B gp120.
(TIF)

S4 Fig SPR analysis of gp120 binding to mAbs specific to the V3 loop and CD4 bs. Anti-human IgG Fc was immobilized on a CM5 sensor chip followed by mAb V3 or CD4bs capture. Various concentrations of A244.AE/6240.B gp120 protein were injected onto the captured mAb V3/CD4bs surface. Sensograms are shown with a single run for each concentration while the kinetics values were computed from at least three independent replicates (**S6 Fig**). (**A**) mAb 2219 vs. A244.AE gp120; (**B**) mAb 2219 vs. 6240.B gp120; (**C**) mAb 2557 vs. A244.AE gp120; (**D**) mAb 2557 vs. 6240.B gp120; (**E**) mAb VRC01 vs. A244.AE gp120; (**F**) mAb VRC01 vs. 6240.B gp120; (**G**) mAb VRC03 vs. A244.AE gp120; (**H**) mAb VRC03 vs. 6240.B gp120.
(TIF)

S5 Fig Sequence alignment of V1V2 loops for CRF01_AE and B using Clustal Omega. The amino acid sequences are highlighted in blue color. Amino acid residues that differ in the alignment are depicted as “.” or “:” while identical amino acids are shown as “*”.
(TIF)

S6 Fig Comparison of kd off-rates between groups receiving monovalent and bivalent gp120 vaccines. (**A**) comparison of kd off-rate for animal groups immunized with MF59 and (**B**) comparison of kd off-rate for animal groups immunized with AH. Differences between groups (monovalent vs. bivalent) were compared using Wilcoxon rank-sum test with confidence level of p<0.05 with significant differences indicated.
(TIF)

S7 Fig Pie chart analysis for proportions of total linear binding response targeting each epitope region. Each pie slice represents the medium binding intensity of the Groups 2, 3, and 6 to the specified epitope, with the sum of intensities to all epitopes (total linear response) of the clade indicated beneath the chart.
(TIF)

S8 Fig Evaluation of virus neutralizing Ab responses in guinea pig sera. Virus neutralizing Ab responses were measured using sera collected at 4 weeks post-4^th^ immunization as described in Methods against: (**A**) Tier-1A subtype CRF01_AE TH023.6; (**B**) Tier-1A subtype B MN.3; (**C**) Tier-1A subtype B SF162.LS; (**D**) Tier-1A subtype C MW965.26. Each symbol represents the ID50 titer for an individual animal with geometric mean for each group indicated by the bar and standard error by the box. Differences between groups (MF59 vs. AH) were compared using Wilcoxon rank-sum test with confidence level of p < 0.05 with significant differences shown where applicable.
(TIF)

S1 Table Identification of glycosylated peptides A244.AE gp120 protein by LC-MS/MS. (TIF)

S2 Table Identification of glycosylated peptides 6240.B gp120 protein by LC-MS/MS. (TIF)

S3 Table Values for group comparisons for peptide array analyses. Values were compared using Wilcoxon rank-sum test, with confidence level of p < 0.05. Tests were not available (NA) when there was no detectible signal in either group being compared.
(TIF)

S4 Table Comparisons of the kd off-rate avidities of immune sera collected at different time points and between groups. Values were compared using Wilcoxon rank-sum test when considering testing between groups at specific time points and Wilcoxon signed-rank test when considering testing between time points at specific groups, with confidence level of p < 0.05.
(TIF)

S5 Table The kd off-rate avidity assessment immune sera collected 4 weeks following each immunization. (TIF)
