# Supplementary figures and images for: Generation and characterization of a bivalent protein boost for future clinical trials: HIV-1 subtypes CR01_AE and B gp120 antigens with a potent adjuvant
Source: PLoS One. 2018 Apr 26;13(4):e0194266. doi: 10.1371/journal.pone.0194266 (PMC5919662; doi:10.1371/journal.pone.0194266)

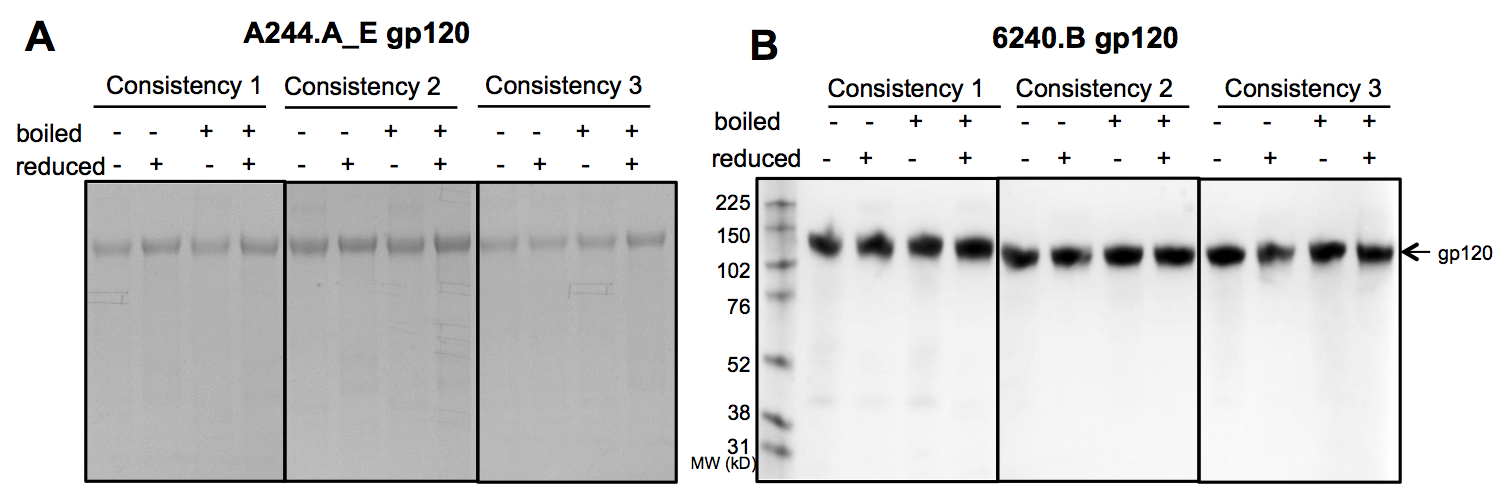

Supplement: S1 Fig — SDS-PAGE of purified (A) A244.AE gp120 protein and (B) 6240.B gp120 protein from three independent rounds of production and purification, respectively. It was noticed that there were several pen marks on some lanes and they were kept with an original image. (TIF) [file pone.0194266.s001.tif]

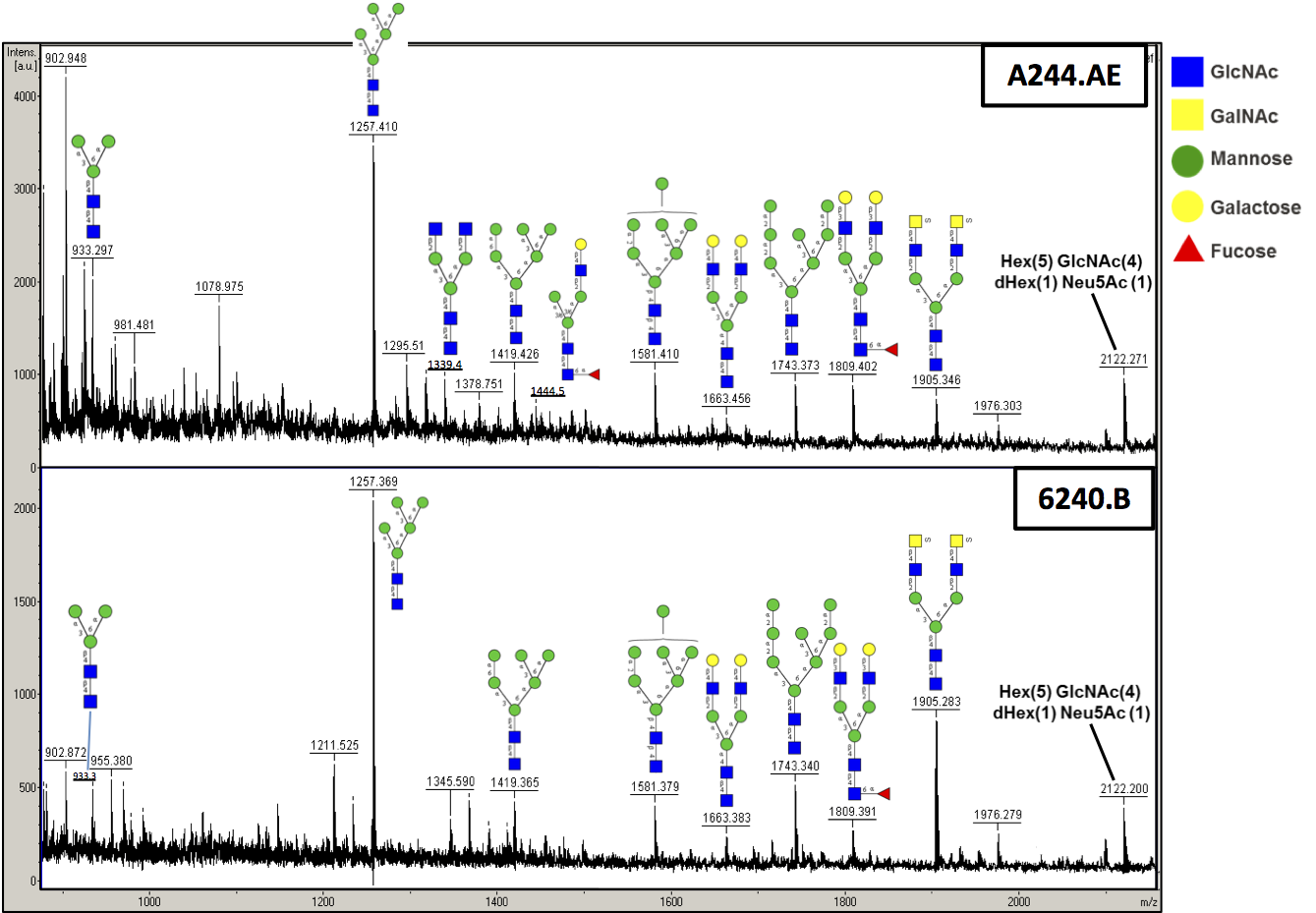

Supplement: S2 Fig — (TIF) [file pone.0194266.s002.tif]

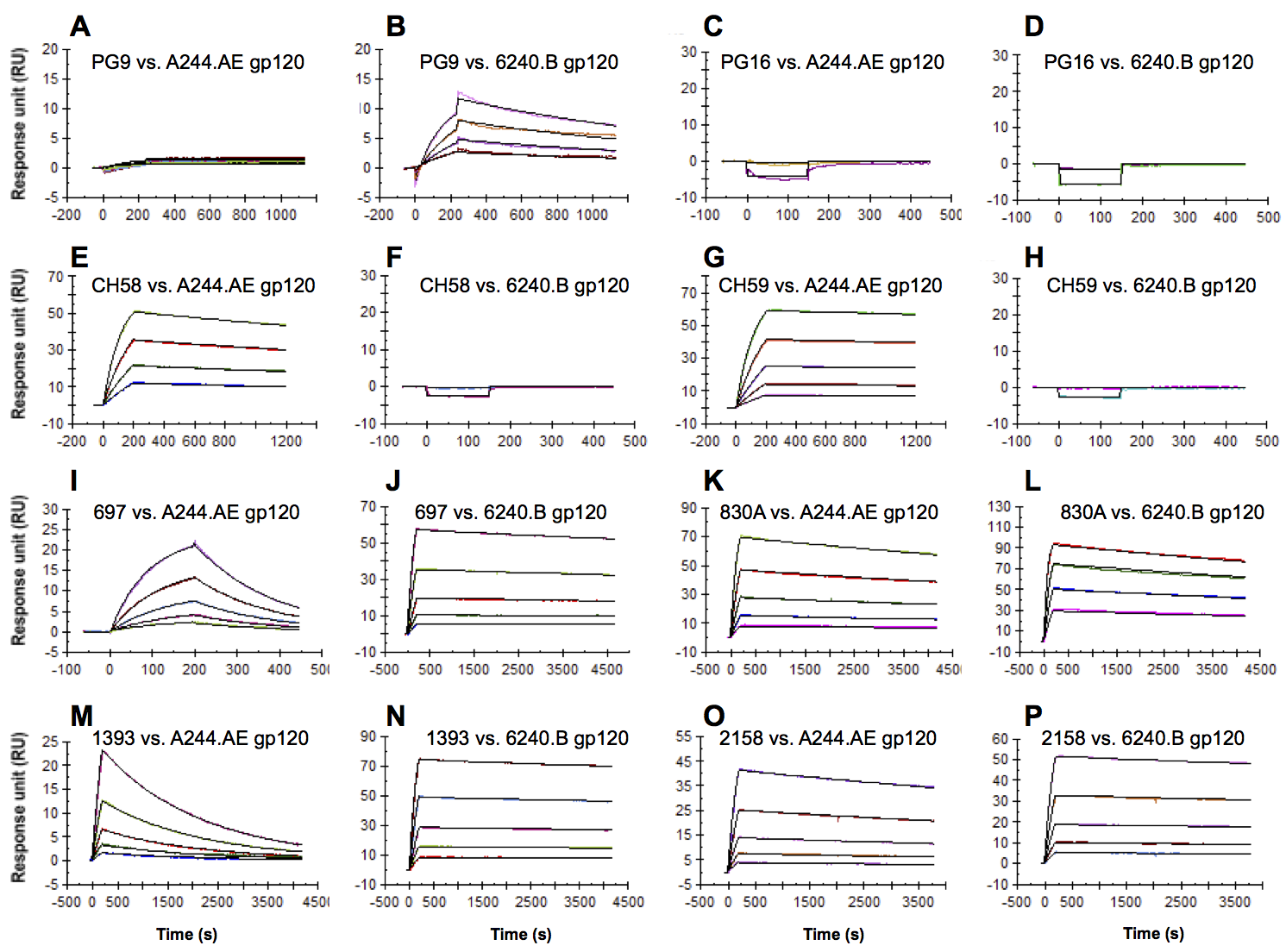

Supplement: S3 Fig — Anti-human IgG Fc was immobilized on the sensor CM5 chip followed by mAb V1V2/V2 capture. Next, various concentrations of A244.AE/6240.B gp120 protein were injected onto the captured mAb V1V2/V2 surface. The sensograms were shown in color codes for different concentrations of gp120 proteins. The kinetics values shown in S1 Table were calculated on an average from at least three replicates. (A) mAb PG9 vs. A244.AE gp120; (B) mAb PG9 vs. 6240.B gp120; (C) mAb PG16 vs. A244.AE gp120; (D) mAb PG16 vs. 6240.B gp120; (E) mAb CH58 vs. A244.AE gp120; (F) mAb CH58 vs. 6240.B gp120; (G) mAb CH59 vs. A244.AE gp120; (H) mAb CH59 vs. 6240.B gp120; (I) mAb 697 vs. A244.AE gp120; (J) mAb 697 vs. 6240.B gp120; (K) mAb 830A vs. A244.AE gp120; (L) mAb 830A vs. 6240.B gp120; (M) mAb 1393 vs. A244.AE gp120; (N) mAb 2158 vs. 6240.B gp120. (TIF) [file pone.0194266.s003.tif]

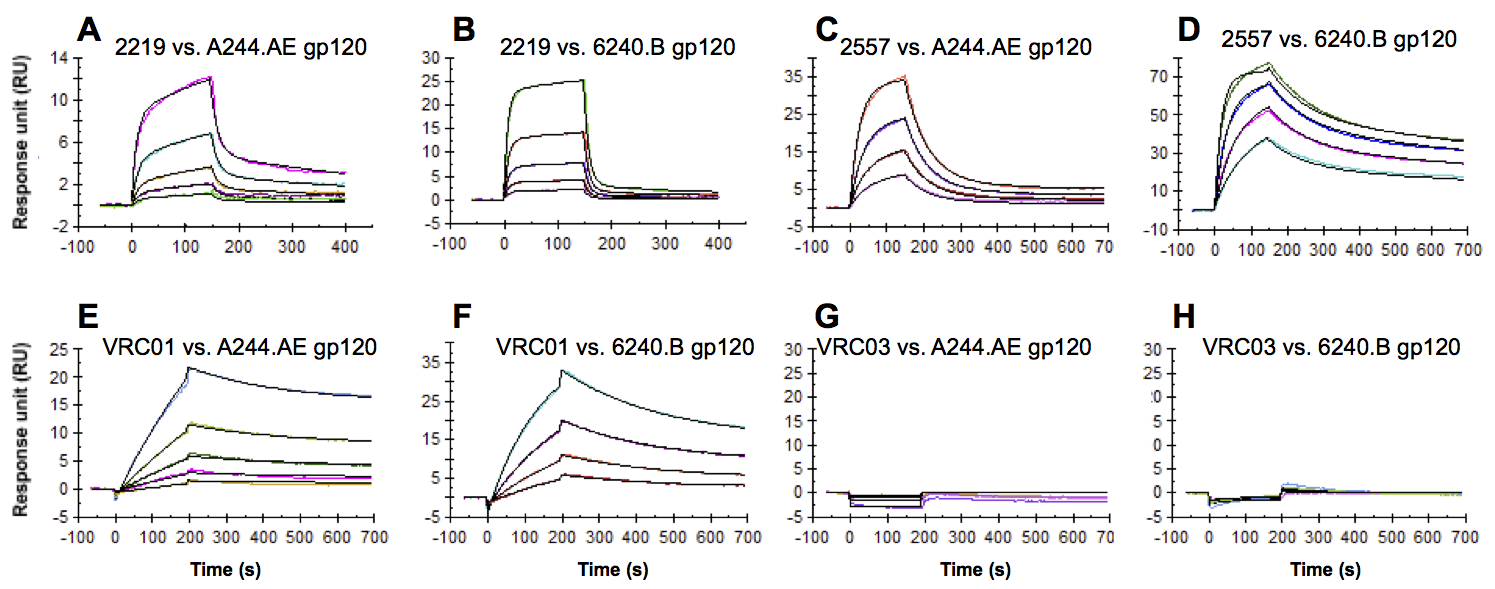

Supplement: S4 Fig — Anti-human IgG Fc was immobilized on a CM5 sensor chip followed by mAb V3 or CD4bs capture. Various concentrations of A244.AE/6240.B gp120 protein were injected onto the captured mAb V3/CD4bs surface. Sensograms are shown with a single run for each concentration while the kinetics values were computed from at least three independent replicates (S6 Fig). (A) mAb 2219 vs. A244.AE gp120; (B) mAb 2219 vs. 6240.B gp120; (C) mAb 2557 vs. A244.AE gp120; (D) mAb 2557 vs. 6240.B gp120; (E) mAb VRC01 vs. A244.AE gp120; (F) mAb VRC01 vs. 6240.B gp120; (G) mAb VRC03 vs. A244.AE gp120; (H) mAb VRC03 vs. 6240.B gp120. (TIF) [file pone.0194266.s004.tif]

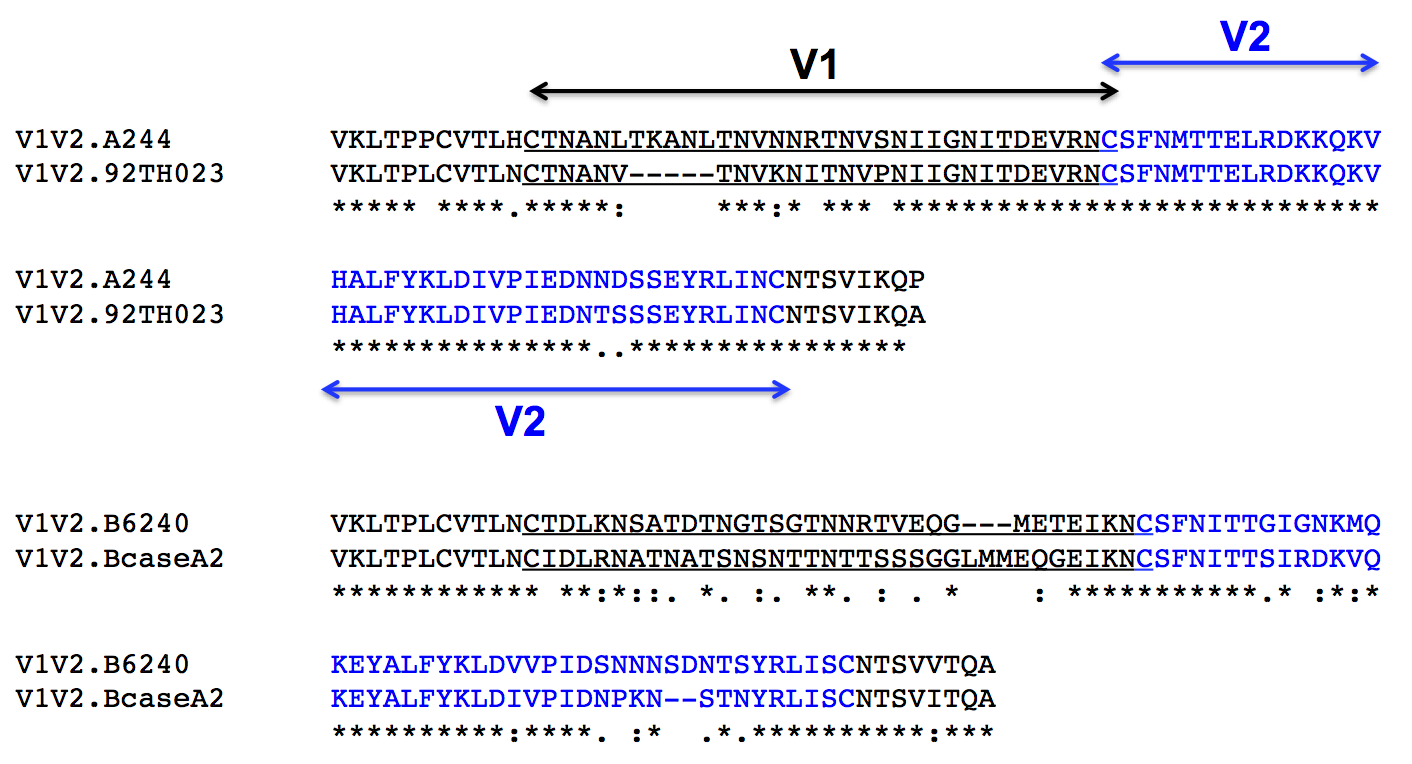

Supplement: S5 Fig — The amino acid sequences are highlighted in blue color. Amino acid residues that differ in the alignment are depicted as “.” or “:” while identical amino acids are shown as “*”. (TIF) [file pone.0194266.s005.tif]

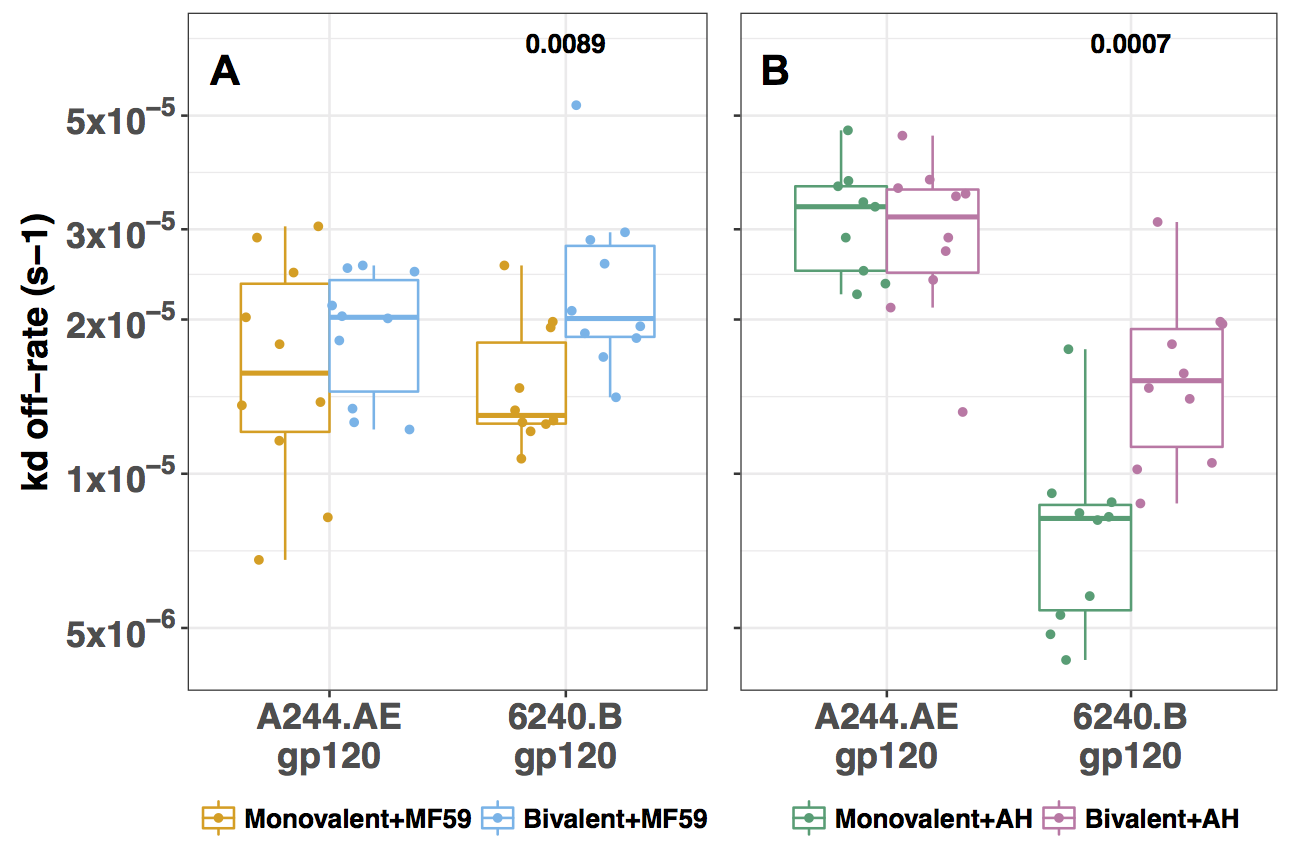

Supplement: S6 Fig — (A) comparison of kd off-rate for animal groups immunized with MF59 and (B) comparison of kd off-rate for animal groups immunized with AH. Differences between groups (monovalent vs. bivalent) were compared using Wilcoxon rank-sum test with confidence level of p<0.05 with significant differences indicated. (TIF) [file pone.0194266.s006.tif]

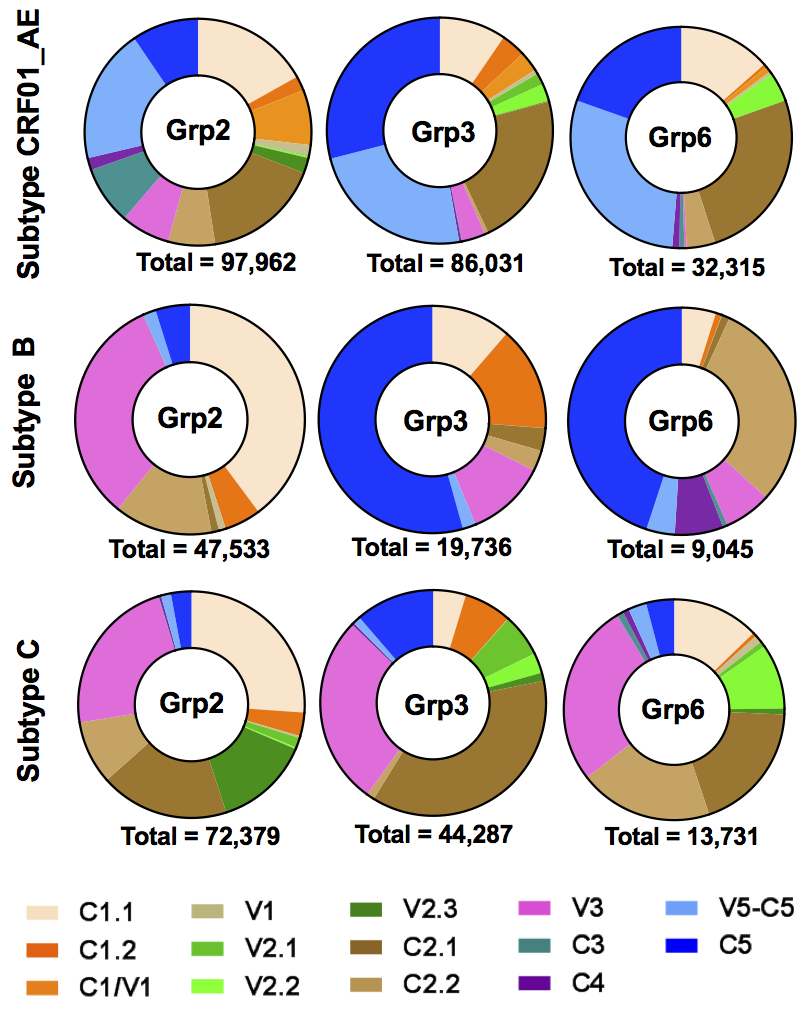

Supplement: S7 Fig — Each pie slice represents the medium binding intensity of the Groups 2, 3, and 6 to the specified epitope, with the sum of intensities to all epitopes (total linear response) of the clade indicated beneath the chart. (TIF) [file pone.0194266.s007.tif]

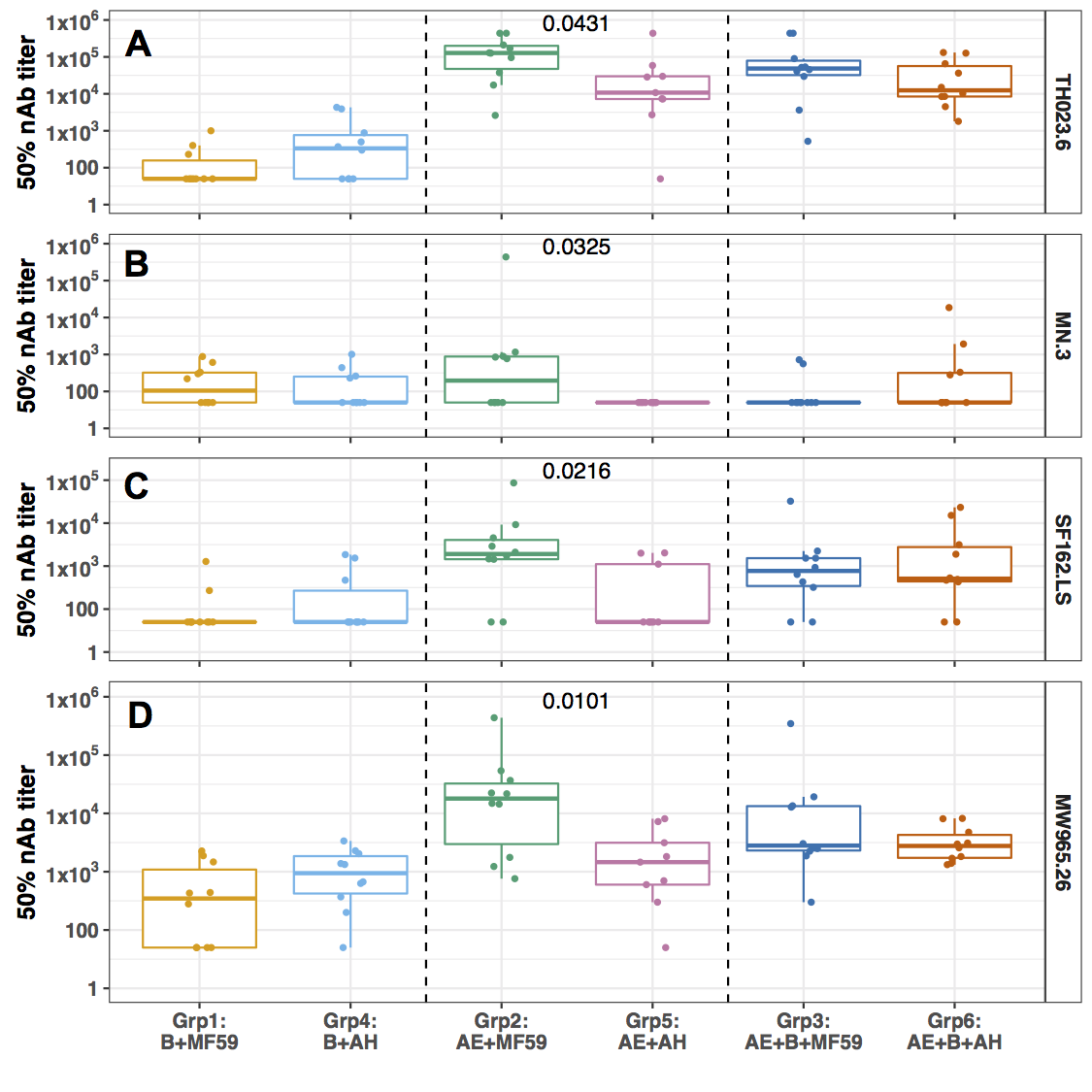

Supplement: S8 Fig — Virus neutralizing Ab responses were measured using sera collected at 4 weeks post-4th immunization as described in Methods against: (A) Tier-1A subtype CRF01_AE TH023.6; (B) Tier-1A subtype B MN.3; (C) Tier-1A subtype B SF162.LS; (D) Tier-1A subtype C MW965.26. Each symbol represents the ID50 titer for an individual animal with geometric mean for each group indicated by the bar and standard error by the box. Differences between groups (MF59 vs. AH) were compared using Wilcoxon rank-sum test with confidence level of p < 0.05 with significant differences shown where applicable. (TIF) [file pone.0194266.s008.tif]

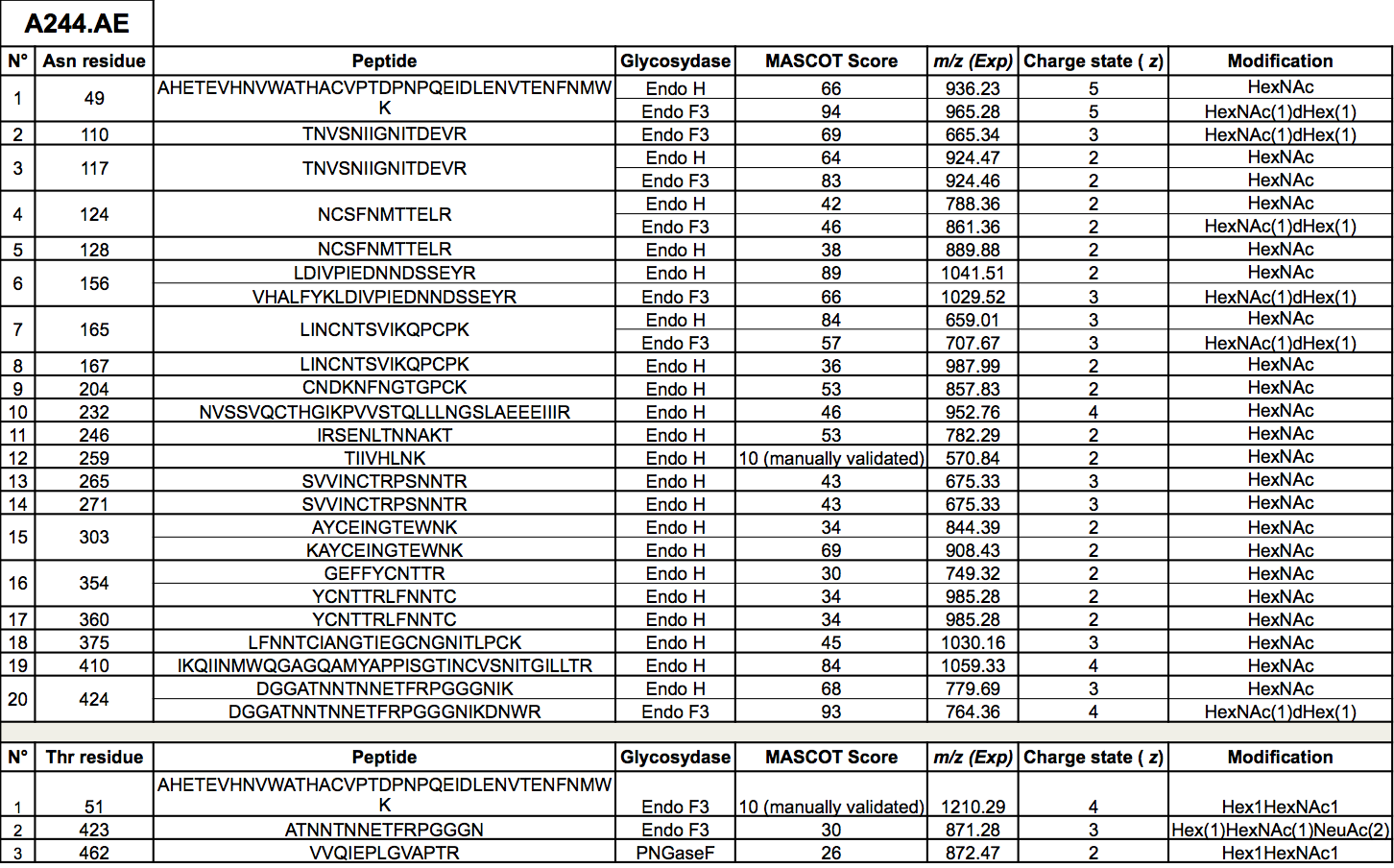

Supplement: S1 Table — (TIF) [file pone.0194266.s009.tif]

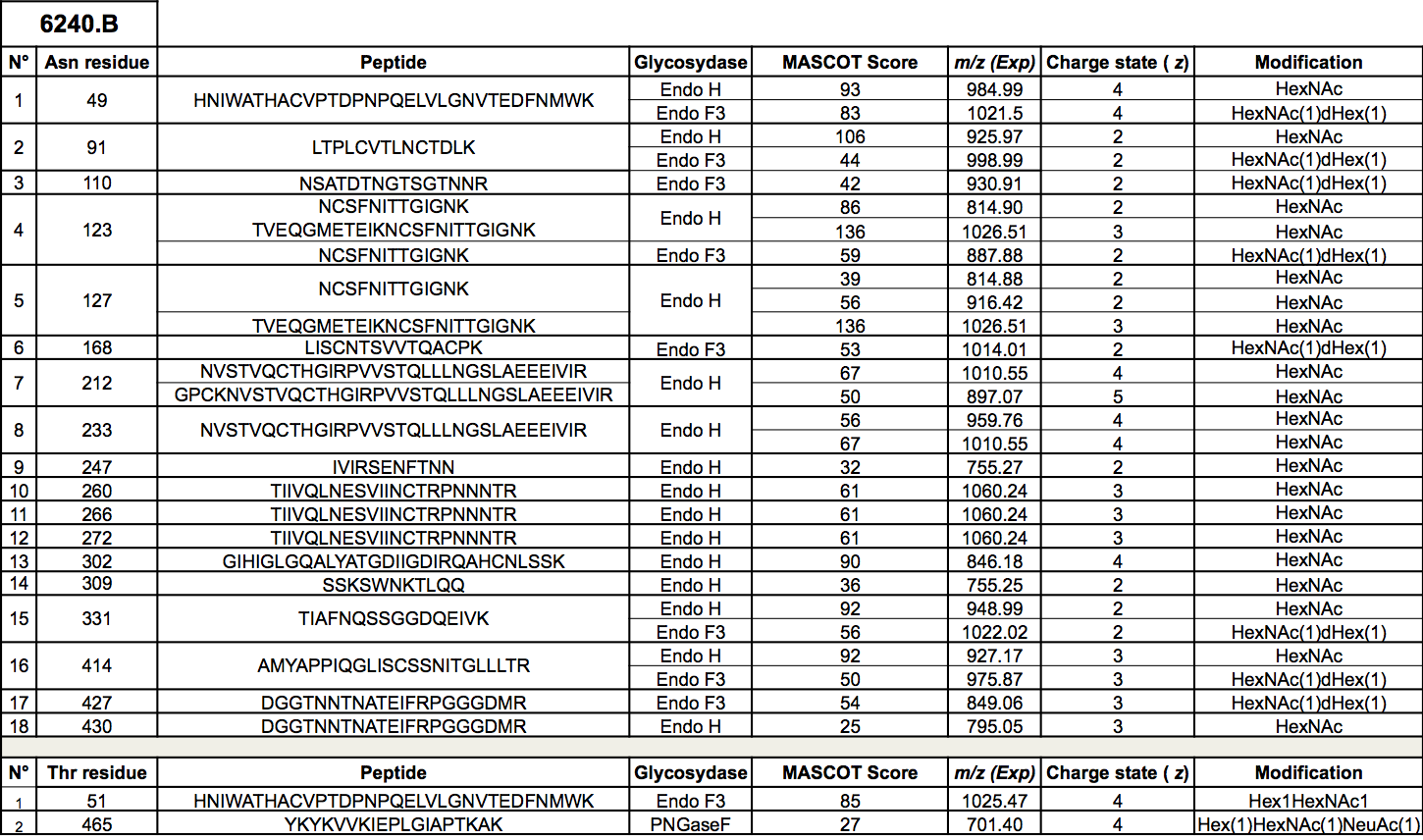

Supplement: S2 Table — (TIF) [file pone.0194266.s010.tif]

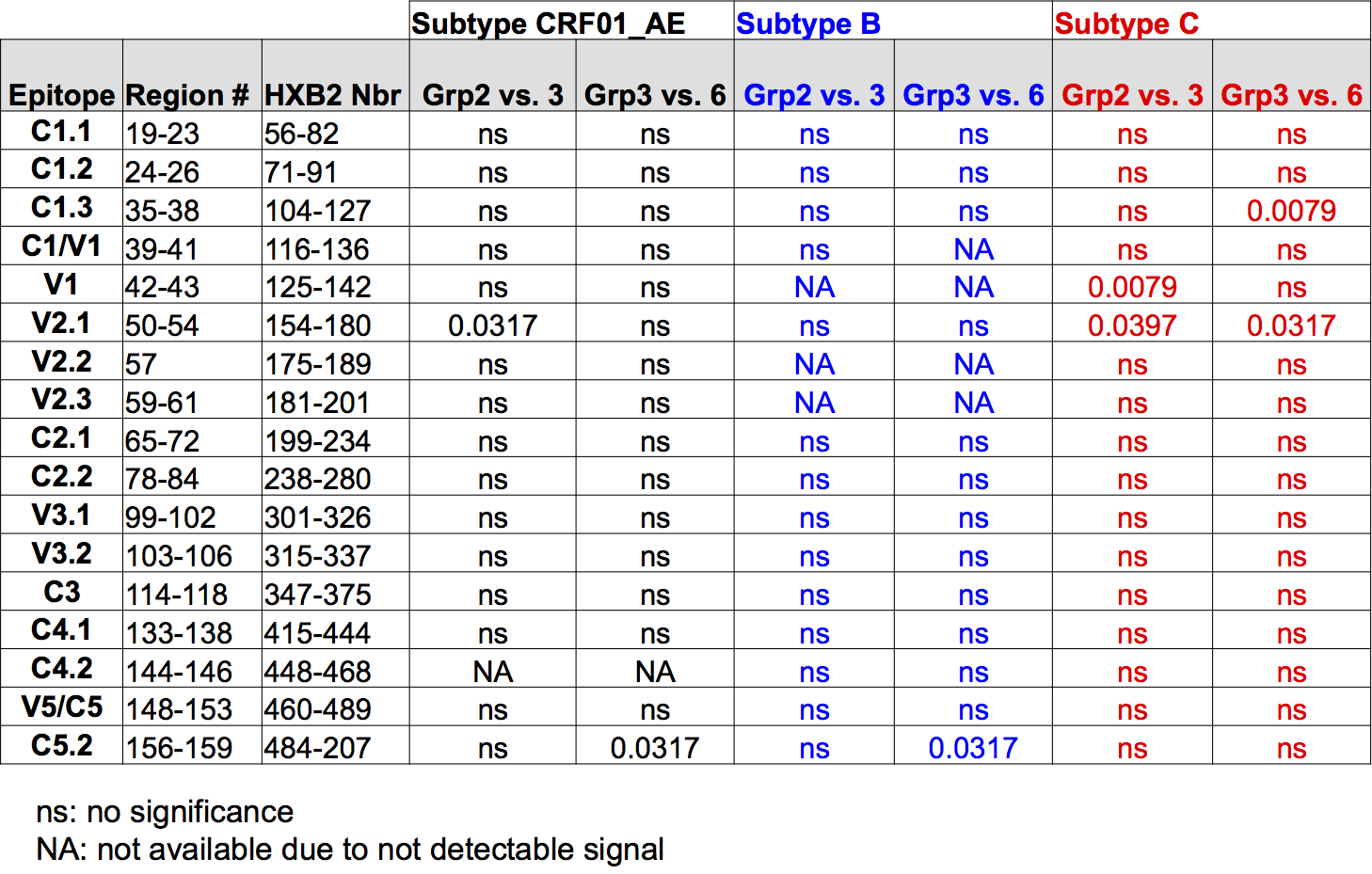

Supplement: S3 Table — Values were compared using Wilcoxon rank-sum test, with confidence level of p < 0.05. Tests were not available (NA) when there was no detectible signal in either group being compared. (TIF) [file pone.0194266.s011.tif]

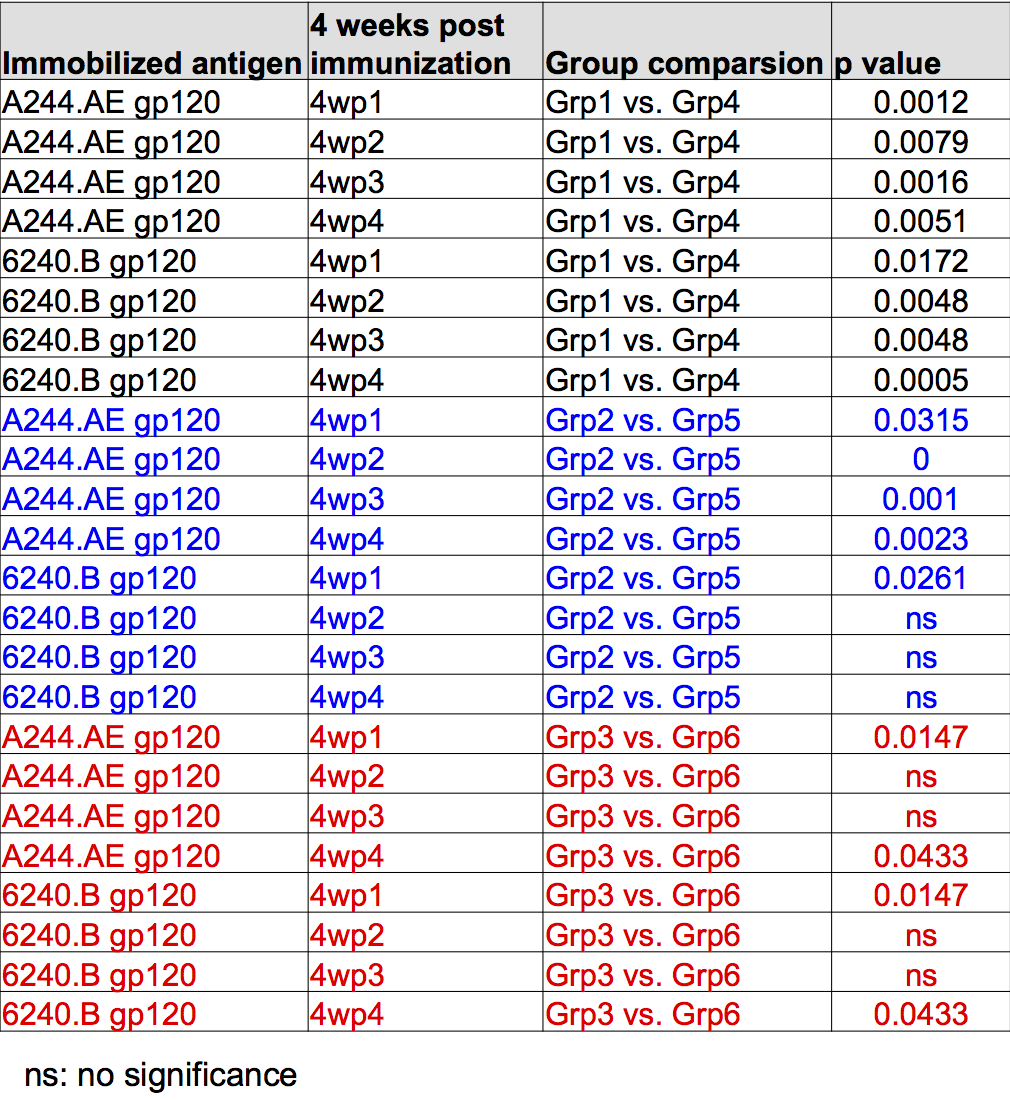

Supplement: S4 Table — Values were compared using Wilcoxon rank-sum test when considering testing between groups at specific time points and Wilcoxon signed-rank test when considering testing between time points at specific groups, with confidence level of p < 0.05. (TIF) [file pone.0194266.s012.tif]

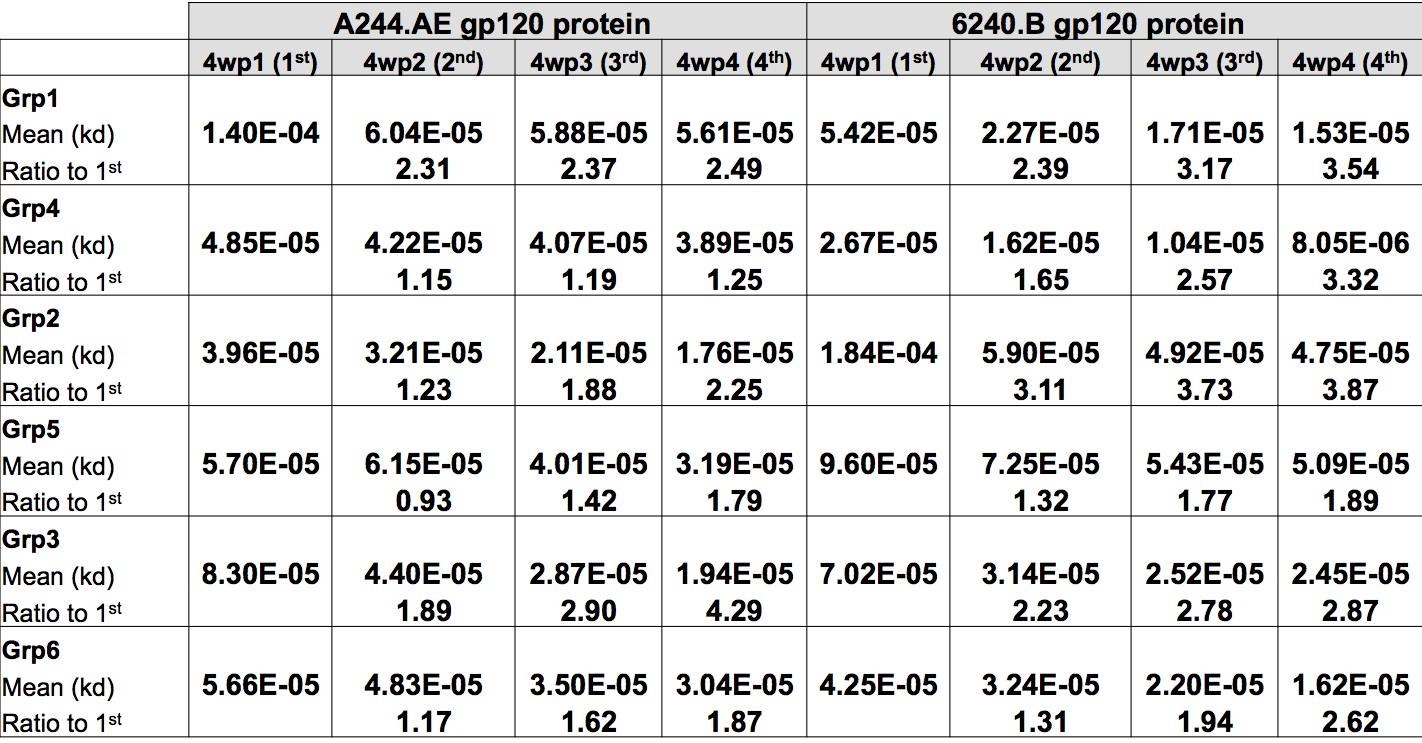

Supplement: S5 Table — (TIF) [file pone.0194266.s013.tif]
